# A multifunctional soft robot for cardiac interventions

**DOI:** 10.1126/sciadv.adi5559

**Published:** 2023-10-25

**Authors:** Jacob Rogatinsky, Dominic Recco, Joseph Feichtmeier, Yuchen Kang, Nicholas Kneier, Peter Hammer, Edward O’Leary, Douglas Mah, David Hoganson, Nikolay V. Vasilyev, Tommaso Ranzani

**Affiliations:** ^1^ Department of Mechanical Engineering, Boston University, Boston, MA 02215, USA.; ^2^ Department of Cardiac Surgery, Boston Children’s Hospital, Boston, MA 02115, USA.; ^3^ Boston University School of Medicine, Boston, MA 02118, USA.; ^4^ Department of Cardiology, Boston Children’s Hospital, Boston, MA 02115, USA.

## Abstract

In minimally invasive endovascular procedures, surgeons rely on catheters with low dexterity and high aspect ratios to reach an anatomical target. However, the environment inside the beating heart presents a combination of challenges unique to few anatomic locations, making it difficult for interventional tools to maneuver dexterously and apply substantial forces on an intracardiac target. We demonstrate a millimeter-scale soft robotic platform that can deploy and self-stabilize at the entrance to the heart, and guide existing interventional tools toward a target site. In two exemplar intracardiac procedures within the right atrium, the robotic platform provides enough dexterity to reach multiple anatomical targets, enough stability to maintain constant contact on motile targets, and enough mechanical leverage to generate newton-level forces. Because the device addresses ongoing challenges in minimally invasive intracardiac intervention, it may enable the further development of catheter-based interventions.

## Teaser

A soft robot increases dexterity, stability, and force transmission for minimally-invasive beating heart interventions.

## INTRODUCTION

As techniques in minimally invasive surgery have matured, the utilization of robots in the operating room has become relatively commonplace (*1*). Surgical robots such as the Da Vinci system (Intuitive Surgical, Inc.) can enhance the operator’s capabilities and enable otherwise difficult or unfeasible procedures (*2*). Benefits of surgical robotic platforms span a variety of applications, from cardiovascular interventions (e.g., precise positioning in cardiac mapping, radiofrequency ablation, and valve repair) (*3*, *4*) to orthopedic surgery (e.g., heightened force generation for hip replacement) (*5*). Surgical robotic platforms have also been developed by multiple research groups to facilitate minimally invasive approaches in hard-to-navigate areas of the body (*6*–*14*).

The delicate nature of biological soft tissue has motivated further research into soft and compliant robots (*15*, *16*). Soft surgical robots aim to provide the same benefits as their rigid counterparts while increasing safety and accessibility and reducing costs (*17*). Key features include minimization of stress concentrations by conforming to delicate structures and the ability to fit into apertures smaller than their nominal size. Exploiting these benefits, soft robots have been successfully introduced for endovascular navigation (*18*–*22*), cardiac operation (*23*–*26*), neurosurgery (*13*, *27*, *28*), lung surgery (*29*–*31*), and endoscopy (*32*–*35*).

The application of robots to interventions inside the beating heart is especially complicated, given the challenging environment and considerable variety of disease etiologies affecting the heart and vasculature. Arrhythmias, valve disorders, coronary artery disease, and heart failure all fall under the umbrella term of cardiovascular disease, making it the leading cause of mortality worldwide (*36*, *37*). To address the growing burden of cardiovascular disease, clinicians have developed transcatheter methods to intervene without the need for open-heart surgery and cardiopulmonary bypass, which may be associated with complications including secondary organ malperfusion and injury (*38*). Minimally invasive beating-heart procedures can reduce operation times, perioperative complications, recovery periods, and costs (*17*, *39*). However, they also face a singular set of challenges specific to the intracardiac environment: (i) a size discrepancy between the vasculature and heart chambers, (ii) a moving workspace generated by cardiac muscle contraction, and (iii) remote tool operation via a distant percutaneous access site.

The discrepancy in scale between the vasculature and heart chambers forces the interventional tools used in the heart to remain small. Interventional catheters must traverse up to a meter or more through peripheral blood vessels, which can accommodate devices up to 8 mm in diameter (i.e., 24 Fr) (*40*–*43*). However, the right atrium (RA), the smallest of the four chambers, has an apicobasal diameter ranging from 34.9 to 58.6 mm (*44*). Limiting the size of the tools to the vascular scale often comes at the detriment of distal force transmission or dexterity once inside the larger intracardiac workspace. In addition, the motile workspace poses a challenge in procedures such as cardiac mapping or radiofrequency ablation, in which interventional tools must maintain stable millinewton-level forces (*45*, *46*). Conversely, procedures such as valve repair require newton-level distal forces despite remote operation making force transmission over the length of the device difficult (*47*–*49*). The beating heart is one of the few anatomical workspaces that present clinicians with a combination of all three listed challenges, making minimally invasive intracardiac intervention especially difficult.

To address these challenges, robot-assisted technologies have been established, especially in the field of electrophysiology (EP) where low contact forces between the catheter and the tissue are required. For example, the Sensei and Magellan (Auris Health Inc.), the Amigo (Catheter Precision Inc.), and the Niobe (Stereotaxis Inc.) are commercially available robotic guide catheters for radiofrequency ablation and cardiac mapping (*50*, *51*). These robotic platforms make use of cable-driven and magnetically actuated catheter sheaths with three degrees of freedom (DoFs), increasing the interventional tool’s achievable task space while remaining small enough to conform with the vascular scale. However, these robots do not account for intracardiac motion. While magnetic actuation results in improved distal force generation, it requires complex and expensive actuation systems to generate external magnetic fields. Cable-driven actuation reduces the need for expensive equipment, but cable friction prompts losses in distal force transmission. Reduced force output is suitable for use in the field of EP, but it is unsuitable for structural heart repairs and other tissue manipulation tasks. Combining the maneuverability of these EP robots with greater stability and force transmission would improve the chances of procedural success in structural repair (*52*), yet there are currently no commercial robots designed for this.

Research prototypes have been developed to further address the aforementioned challenges. Several groups have addressed the vascular size constraint with small and dexterous catheters that can navigate tortuous paths and perform interventional procedures (*19*, *21*, *53*, *54*). While the actuation methods vary across these devices, they maintain an overall flexibility that allows for easy vascular navigation. Another set of devices addresses the motile environment with a variety of methods. One group designed a robot to operate on the outer surface of the heart. The robot uses mechanical bracing against the local anatomy to mitigate the effects of cardiac motion and was able to perform myocardial injections (*25*). Cardiac motion has also been addressed by shape-locking against blood vessels and anchoring through or onto intracardiac surfaces (*55*). Such systems have been shown to improve stability while guiding catheters through the vasculature or atrial septum (*56*, *57*). Alternatively, tool stability inside the beating heart has been addressed via motion compensation algorithms. For example, force feedback combined with motion compensation via three-dimensional (3D) ultrasound has allowed users to compensate for rhythmic heart motions and maintain tissue contact forces (*47*, *48*, *58*, *59*). Last, the challenge with force transmission during remote operation has been addressed by focusing on distal stiffness using concentric tubes or rigid structural backbones to generate forces compatible with the requirements for reconstructive procedures such as valve repair (*8*, *49*, *60*).

Despite these advances, current devices struggle to reconcile a small size requirement for percutaneous access with preservation of distal dexterity, stability, and force transmission. This encumbers standard procedures in EP and interventional cardiology, and may preclude the advancement of minimally invasive approaches to complex reconstructive procedures such as valve repair. There is a need for devices that can dexterously maneuver within the heart and guide existing instruments toward a variety of anatomical targets while maintaining stability and distal force transmission.

**Fig. 1.**
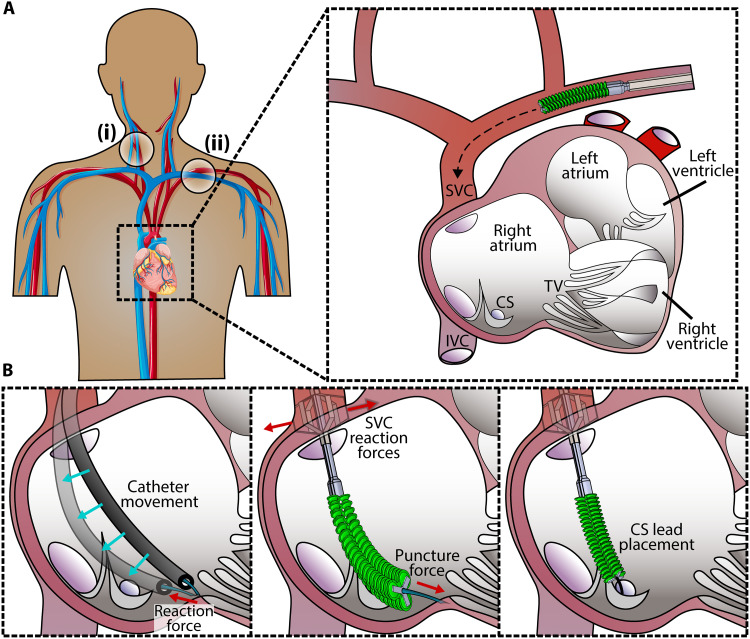
Clinical workflow for the demonstrated device applications. (**A**) The device moves toward the right atrium through the superior vena cava via one of two peripheral access sites: (i) the right internal jugular vein or (ii) the left subclavian vein. (**B**) Left: Illustration of current challenges with conventional catheters, where intrinsic flexibility makes it challenging to apply forces and accurately control position. Middle: The proposed device can self-stabilize against the superior vena cava to minimize losses in distal force generation. This stabilization, as well as the soft robotic tip, enables the user to perform procedures such as tricuspid valve annular anchor implantation and coronary sinus pacemaker lead placement. The robot is illustrated deploying an anchor into the tricuspid valve annulus. Right: The robot is illustrated guiding a guidewire into the coronary sinus.

Here, we demonstrate a multifunctional soft robotic catheter for intracardiac use to address the size discrepancy, moving workspace, and remote access challenges in beating-heart surgery. The proposed robot is small enough to access the heart via the vasculature (Fig. 1A), but its ability to expand and exploit the larger cardiac workspace allows for stability, dexterity, and force output despite a motile environment and distant operation. It is also capable of stabilizing against the vasculature proximal to the heart and actively steering instruments within the heart (Fig. 1B). It consists of millimeter-scale deployable mechanisms that address the scale discrepancy, moving workspace, and distant tool operation challenges. Specifically, the robotic platform features a stabilization component that combines semirigid and flexible materials into a structure that expands and braces at a venous entrance to the heart. It was designed to provide leverage to an active steering component while minimizing physiological stresses such as blood pressure gradient in access vessels and vascular damage. The active steering component, composed of entirely soft materials, allows the fluid used for actuation and the flexibility of its constituent materials to provide damping against external forces. Its kinematics were programmed to allow the user to position interventional tools at any point in the intracardiac workspace.

We demonstrate the multifunctional robotic catheter by simulating two exemplar procedures in the RA: an electrophysiological procedure [i.e., coronary sinus (CS) pacemaker lead placement] and a reconstructive procedure [i.e., tricuspid valve (TV) anchor implantation for annuloplasty procedures]. These procedures were chosen because of the mounting evidence supporting the need for greater attention to right side diseases (*61*, *62*), and the Supplementary Materials include an extended discussion on their clinical relevance and current challenges. Together, CS cannulation and TV annuloplasty require high dexterity, stability, and force transmission to address the three major challenges of intracardiac intervention. We introduce the design and manufacturing of the robot and validate its performance with respect to these challenges, both in vitro and ex vivo.

## RESULTS

We introduce a soft robotic platform for minimally invasive intracardiac beating-heart intervention, consisting of a deployable stabilization mechanism and a collapsible soft manipulator. The combination of stabilization and robotic tool guidance enables versatility in performing clinical tasks such as CS lead placement and less standardized procedures such as TV repair. It is further capable of guiding existing interventional instruments toward a variety of targets in the RA via a 2-mm (i.e., 6 Fr) working channel that runs through its entire length. In this way, it provides a direct connection between the user and the anatomic target in the heart. The dimensions of the central channel and the rest of the robot are detailed in section S9.

The robotic platform features a shape-locking, expandable stabilization mechanism manufactured with a 2D paradigm used in the fabrication of millimeter-scale mechanisms (*57*, *63*–*65*). This stabilization mechanism is capable of shape-locking against the proximal superior vena cava (SVC) without puncturing it, preventing undue harm to the delicate SVC vasculature and the sinoatrial node. In addition, the local stabilization shifts the fulcrum of the catheter from the peripheral access point to the anatomical target. Moving the fulcrum toward the target site provides more mechanical leverage to the surgical tool, improving distal force transmission compared to nonstabilized instrumentation (Fig. 1B). This improved force transmission and stability at the distal end of the robot is independent of how far the entry access is. However, as moving the fulcrum of a beam toward one end decreases that side’s lever arm, so too does moving the fulcrum of a catheter toward its distal tip decrease its mobility. Without the addition of an active steering component, distal stabilization would notably reduce the catheter’s reachable intracardiac task space.

We therefore integrated a multi-DoF soft robot to expand the device’s task space. The soft robot was designed to collapse to a diameter of 24 Fr, allowing it to fit in peripheral blood vessels (*40*–*43*), and then expand once inside the heart to increase its surface area and force output. The soft robot further addresses the challenge of instability in a motile environment through its inherent material damping. It is capable of conforming to the heart wall’s motions, thereby maintaining contact with a moving target during a simulated procedure.

### Endovascular stabilization

**Fig. 2.**
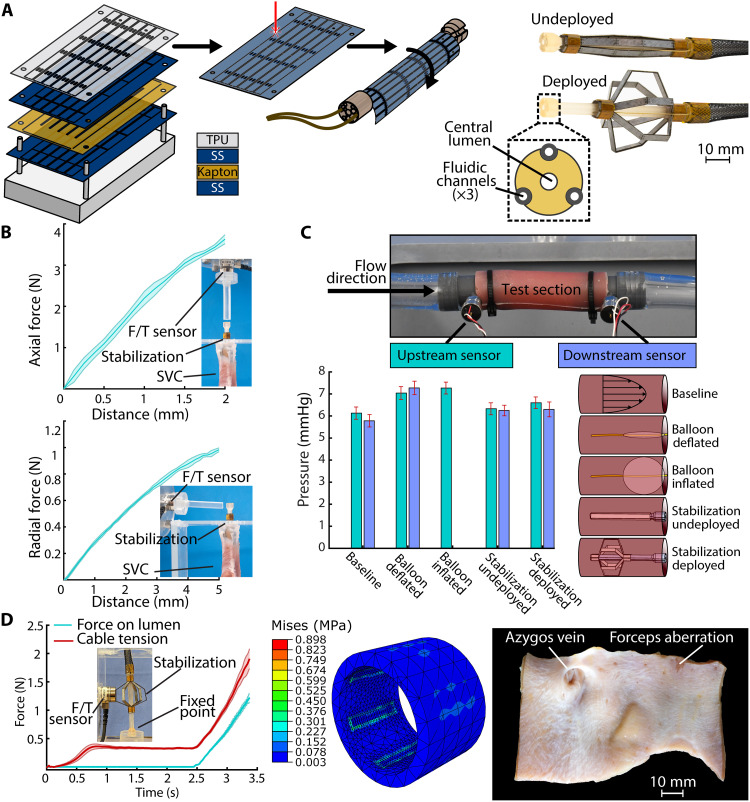
Stabilization mechanism design, fabrication, and characterization. (**A**) Alternating layers of stainless steel, polyimide, and thermoplastic polyurethane (TPU) are laser cut, stacked, and adhered with bioadhesive. The laminate is wrapped around a 3D printed tube. (**B**) Reaction forces on the stabilization mechanism deployed in an explanted porcine superior vena cava (SVC) after being indented *n* = 5 times each in the axial (top) and radial (bottom) directions. (**C**) Testing setup, results, and schematic representations of each test case for measuring the downstream pressure caused by the stabilization mechanism. (**D**) Force generated on a lumen by the stabilization mechanism relative to Bowden cable tension (left) and ABAQUS model showing stress concentration areas for the maximum experimental force (center). Right: Microscope picture of the SVC endothelium after 4 hours of repeated stabilization deployment.

We manufactured the stabilization component using a layer-by-layer process shown in Fig. 2A. First, we laser cut individual sheets of semirigid spring steel and flexible polyimide film. These layers were adhered using a biocompatible pressure-activated adhesive to create a laminate with two layers of spring steel sandwiching a single layer of polyimide. The laminate was then coated with a soft thermoplastic polyurethane (TPU) layer before being subjected to a release cut from the sacrificial material. The primary purpose of this TPU coat is to soften the edges of the steel joints to reduce the stress concentrations imparted on the SVC. The resulting laminate consists of six flexure joints oriented parallel to each other and connected at either end. Once released from the sacrificial material, the laminate was wrapped around a stereolithography (SLA)-printed multilumen tube using biocompatible resin (Fig. 2A). The multilumen tube accommodates the three fluidic tubing lines used to actuate the soft robot and two Bowden cables used to deploy the stabilization mechanism. The resulting stabilization assembly expands to 32 mm in diameter and collapses to a diameter of 8 mm when undeployed. The TPU coat serves a secondary purpose during deployment as a store for elastic energy. This provides a restorative force that causes the stabilization mechanism to default to its collapsed state during nondeployment. When deployed, the stabilization mechanism assumes a wireframe-like structure with a cumulative cross-sectional area ≤50% that of the SVC, allowing continued blood flow through the vein. The stabilization can also be quickly collapsed for removal of the robot or reorientation of the soft manipulator’s base.

We first tested the stabilization mechanism’s ability to generate mechanical leverage for the soft robot. To do this, we deployed the stabilization mechanism in an explanted section of porcine SVC. We then indented its tip by a fixed distance in the radial and axial directions, measuring the force required for the given displacement. The stabilization mechanism withstood 0.98 ± 0.02 N of radial force at 5 mm of displacement and 3.63 ± 0.11 N of axial force at 2 mm of displacement (Fig. 2B).

We then verified the stabilization mechanism’s physiological compatibility with respect to the flow pressure and stress concentrations resulting from its deployment. Given that pressure in the RA is ≈5 to 8 mmHg, an extended pressure buildup can lead to hemorrhage in smaller cranial blood vessels (*66*). In addition, the saphenous vein, which is similar in mechanics to the SVC, experiences circumferential failure at a stress of 1.8 MPa (*67*). We use these pressure and stress values from the literature as benchmarks to guide these safety validations.

The pressure drop caused by the stabilization mechanism was experimentally determined by activating it in a section of silicone tubing with a pressure sensor on either end to measure water flow pressure (Fig. 2C). The baseline pressure values at the upstream and downstream sensors were 6.13 ± 0.28 mmHg and 5.78 ± 0.28 mmHg, respectively. A generic balloon catheter was then tested as a control in its deflated and inflated states. The deflated balloon catheter’s pressure values were 7.05 ± 0.29 mmHg upstream and 7.27 ± 0.30 mmHg downstream, while the values when inflated were 7.26 ± 0.27 mmHg upstream and zero downstream. This drastic gradient arose because the inflated balloon completely cut off flow from the downstream pressure sensor. Last, the stabilization mechanism was tested in its nonactuated and actuated states. The collapsed stabilization mechanism’s pressure values were 6.33 ± 0.27 mmHg upstream and 6.25 ± 0.24 mmHg downstream, while the values when deployed were 6.60 ± 0.26 mmHg upstream and 6.30 ± 0.34 mmHg downstream. In both cases, the absolute pressure values range from 3.3 to 9.0% higher than the respective baseline values, and the pressure drops do not exceed the baseline pressure drop. Further tests were conducted to assess the effect of the full robot on the fluidic resistance, demonstrating negligible obstruction to the blood flow caused by the robot (see section S12).

To determine the device’s safety when stabilizing against the SVC, we first determined the force that it could apply on a lumen when the actuating Bowden cable was pulled 15 mm, the length required for the stabilization to fully expand (Fig. 2D). This test was performed twice: once with the central axis of the stabilization mechanism fixed and once with the device deployed in a silicone tube to allow for radial translation. We then simulated these scenarios in ABAQUS using the experimental force results as input, yielding theoretical stress concentrations on the SVC as output. We approximated the SVC’s mechanical properties using those of the porcine saphenous vein (*68*).

At a maximum Bowden cable tension of 1.91 N, the stabilization mechanism generated 1.21 N of radial force. This radial force yielded simulated maximum stress concentrations of 0.898 MPa on the vein. This stress value represents a theoretical maximum based on idealized testing conditions with a rigid force sensor and the central axis of the stabilization mechanism fixed in place. In a more realistic scenario, the stabilization mechanism’s central axis would not be fixed, and the elasticity of the vein would dissipate some of the stress resulting from mechanical strain. To emulate this, we performed a similar test in which the stabilization mechanism was placed in a silicone test section, allowing the central axis to translate radially upon contact with the walls and force sensor. In this case, a maximum Bowden cable tension of 4.99 N yielded only 0.43 N of radial force from the stabilization mechanism. We found a simulated maximum stress concentration of 0.212 MPa on the vein. Given that the saphenous vein fails circumferentially at 1.8 MPa, the applied forces would not induce failure (*67*).

The theoretical results were verified experimentally by deploying the stabilization mechanism multiple times in a section of porcine SVC during one 4-hour ex vivo test for CS cannulation. We dissected the SVC upon completion of testing to analyze the endothelium under a microscope. There were no visible signs of damage from the stabilization mechanism (Fig. 2D). These empirical results corroborate the simulation results despite the linear elastic SVC material model and lack of convergence study. Such analyses would be necessary if multiple designs or complex loading cases were to be compared. However, given the experimental safety validation, the assumptions made regarding material models and mesh size can be considered sufficient for approximation.

### Soft robotic guidance

We use a class of soft robotic manipulator called the stacked balloon actuator (SBA) (*29*, *69*, *70*) to robotically guide surgical instrumentation (fig. S2). The SBA architecture provides several advantages, including a large expansion ratio and high force generation. Because these actuators are fabricated by subjecting a 2D laminate of TPE (thermoplastic elastomer) and polytetrafluoroethylene (PTFE; Teflon) to heat and pressure, they can be fully collapsed in the axial direction (fig. S2, A and B). However, depending on the number of layers used, the inflatable balloon geometry can expand to an arbitrarily large length. Here, our laminate contains three stacks of 20 balloons positioned radially around a 6-Fr central channel, such that each chamber’s longitudinal axis falls on the vertex of an equilateral triangle with side length ≈7 mm (fig. S2C). This yields a soft SBA that can expand from a deflated thickness of ≈1 mm to a fully inflated height of nearly 4 cm without compromising the cross-sectional area of the central channel. In addition, prior works have shown that the SBA can generate forces of up to several newtons in bending and extension when actuated to 100 kPa (*69*, *70*).

While the SBA’s fabrication causes it to naturally collapse along its main axis, delivery through the vasculature necessitates the additional ability to radially collapse to a diameter of 24 Fr (fig. S2B). To achieve this radial collapse, the actuator was inflated equally in all three chambers to fully elongate it. The tip was then manually fixed to maintain a constant length, while vacuum was applied to the chambers. The applied vacuum would normally cause the chambers to fold axially according to their embedded geometry, but the fixed length caused them to buckle and collapse radially. We demonstrate that a three-chamber SBA with a nominal diameter of 15 mm can radially collapse to a minimum diameter of 4.5 mm. We used this method of radial SBA collapse during deployment in clinical tests, as shown in movie S2. Experimental characterizations of the soft SBA were performed as part of the fully integrated robotic platform.

### Integrated robotic platform

**Fig. 3.**
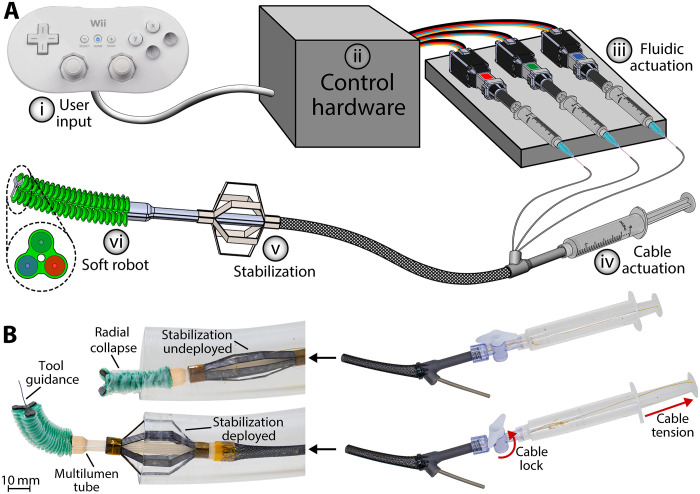
Integrated robotic platform overview. (**A**) The device is operated with a six-step workflow: (i) The user controls the soft robot’s fluidic chambers with a Nintendo Wii Classic controller. (ii) User inputs are interpreted by an Arduino Leonardo microcontroller. (iii) The microcontroller sends signals to three linear motors (Actuonix, S20), which actuate their respective water-filled syringes. (iv) The user deploys the stabilization mechanism by pulling on a syringe plunger, which generates tension in a Bowden cable. (v) The stabilization mechanism deploys against the superior vena cava (SVC) as a result of step (iv). (vi) The soft robot is controlled as a result of steps (i) to (iii). (**B**) The soft robotic component is capable of radially collapsing to the device’s diameter of 8 mm, allowing it to fit through a 24-Fr lumen as it moves through the vasculature. In addition, the termination of the Bowden cable for the stabilization mechanism routes through a Luer lock valve and into a syringe, allowing the user to lock the stabilization after pulling the cable.

An overview of the integrated soft robotic platform is presented in Fig. 3. The robot was assembled by first connecting the soft manipulator to the stabilization mechanism’s 3D printed multilumen tube. A single layer of stainless steel was laser cut, and its surface was roughened to make the connection between the soft manipulator’s base and the multilumen tube more robust. The manipulator was adhered to the steel base using bioadhesive, and the steel base was similarly adhered to the multilumen tube. The three fluidic tubing lines that supply each manipulator chamber were routed through three half-channels in the multilumen tube, allowing them to remain hidden underneath the stabilization laminate (Fig. 2A). This assembly was then attached to a 30-cm-long section of mesh lining, which concealed the three fluidic tubing lines and the two Bowden cable sheaths from the stabilization mechanism. This mesh lining additionally concealed a length of PTFE-lined catheter tubing, which constitutes the device’s central channel. The central channel is used to deploy conventional surgical instrumentation such as guidewires and catheters through the robot.

At the user’s end of the interventional robot, a custom SLA-printed part split the Bowden cable terminations from the central channel and fluidic tubing lines (Fig. 3A). The central channel terminated freely, allowing the user to insert a tool through it, while the fluidic tubing lines were connected to custom syringe pumps. The Bowden cables were routed into a syringe with a Luer lock valve connector. By pulling the syringe and locking the valve, the Bowden cables are clamped, thereby locking the stabilization mechanism in its deployed position (Fig. 3B).

**Fig. 4.**
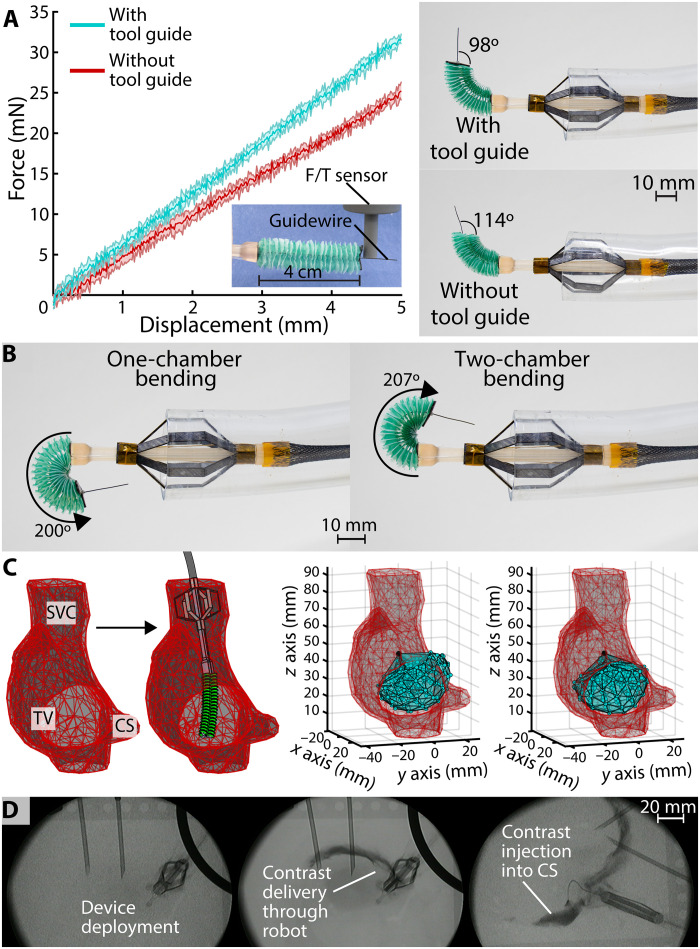
Integrated robot main characterizations. (**A**) Effect of addition of the tool guide on the stiffness (left) and on the angle of deployment of a 0.35-mm guidewire. (**B**) Soft manipulator bending beyond retroflection, with a difference of 3.5% between the (left) one-chamber and (right) two-chamber bending cases. (**C**) Left: A 3D model of the right atrium (RA) generated from a computed tomography (CT) scan of the heart. Right: 3D model of the RA with overlaid task space of the soft manipulator. The base of the point cloud was rotated to simulate a clinician rotating the stabilization mechanism during deployment. (**D**) Pictures showing the soft robotic platform visualized under fluoroscopy while deployed in the RA of an ex vivo porcine heart. The radiopaque stabilization mechanism and tool guide allow device localization in relation to anatomy like the coronary sinus (CS).

We mounted a 2D tool guide on the tip of the soft manipulator to increase the system’s stiffness and accuracy when guiding an interventional tool (Fig. 4, A and B). The tool guide was manufactured using the same principles as the stabilization mechanism, wherein layers of stainless steel, polyimide, and TPU were precut, adhered, and released on a 5-W ultraviolet (UV) laser (fig. S3). Three unidirectional flexure joints allow the tool guide to fold inward during the soft manipulator’s radial collapse (Fig. 3B). Catheter tubing spanning the length of the device was adhered to the tool guide’s central hole, thereby connecting the user end with the tool end of the device.

We tested the combined stiffness of the device’s distal components by inflating the manipulator to 4 cm in extension and inserting the stiff end of a 0.35-mm guidewire (Fig. 4A). With the mounted tool guide, the assembly had a stiffness of 6.3 mN/mm. Without the tool guide, the guidewire had more freedom to move in the manipulator’s central channel, decreasing the stiffness to 5.0 mN/mm (Fig. 4A). In addition, when the manipulator was actuated to 90° in bending, the tool guide maintained the flexible end of the guidewire at an angle of 97.8° from the horizontal (Fig. 4A). Without the tool guide, the guidewire was free to bend 114.2° from the horizontal. Overall, the tool guide provided a 26% increase in stiffness to the guidewire and a 67% reduction in bending error compared to the case without it.

#### Soft manipulator task space

We demonstrated the extremes of the soft manipulator’s task space with the flexible end of a 0.35-mm guidewire inserted through its working channel (Fig. 4B). In the case where one chamber was inflated and two were kept deflated, the manipulator could bend to 200°. In the two-chamber bending case, it could bend to 207°, 3.5 % higher than the one-chamber bending case. In addition, the soft manipulator demonstrated a radius of curvature of 7.6 mm in both cases. Both radii were calculated by measuring the diameter of the circle circumscribed by the manipulator’s central lumen using fiducial markers and image postprocessing.

The task space of the soft manipulator was experimentally determined and compared with a model of the RA. The anatomical model of the RA, SVC, and CS was created by segmenting a computed tomography (CT) scan of a heart in Mimics Innovation Suite (Materialise) (see Fig. 4C). The resulting SLA mesh represents the internal geometry of the segmented anatomy. The task space was overlaid in the RA mesh, demonstrating the robot’s ability to position the tool guide in clinically relevant locations including the CS and TV annulus (Fig. 4C). The resulting point cloud covers the TV annulus and CS ostium, demonstrating sufficient maneuverability within the RA. In addition, this task space is subject to change based on the operator’s positioning of the device during stabilization deployment. Should the user wish to position the base at a different angle or position, they can simply manipulate the device as they would a conventional catheter before deploying the stabilization mechanism and locking the orientation and position.

The task space was further compared with two theoretical models, described in the Supplementary Materials. The theoretical models are based on two analytical relationships between actuator volume and length, one based on geometrical considerations and one extrapolated from experimental data. These relationships were integrated with constant curvature kinematics to determine a theoretical tip pose for comparison with the experimental task space. The constant curvature assumption allows us to create a computationally efficient forward kinematic model that predicts the tip pose with a median error of 19% and interquartile range (IQR) of 11% (fig. S1). In addition, the median and IQR model error decreases as the arc length of the soft manipulator increases. Because the anatomical targets of interest in the RA (i.e., TV annulus and CS ostium) are at least 25 mm from the base of the soft robot, this trend means that the model’s accuracy is better the closer the soft manipulator gets to its target. For backbone arc lengths of ≥25 mm, the median error is 17% with an IQR of 4%.

#### External imaging compatibility

The device was deployed in an explanted porcine heart and visualized under x-ray fluoroscopy to demonstrate its potential visibility under external imaging (Fig. 4D and fig. S6C). The stabilization mechanism and tool guide are both radiopaque because of their constituent steel layers, making them visible in these images. Direct visualization was used to guide the manipulator toward the CS and cannulate it with an angiography catheter, while fluoroscopy capture was taken through the cardiac tissue of the ex vivo sample. Five mililiters at a time of ioversol injection contrast (350 mg/ml; Liebel-Flarsheim Company LLC) was introduced at a rate of 1 ml/s through a catheter in the robot’s central channel, allowing visualization of the CS.

#### Force transmission

**Fig. 5.**
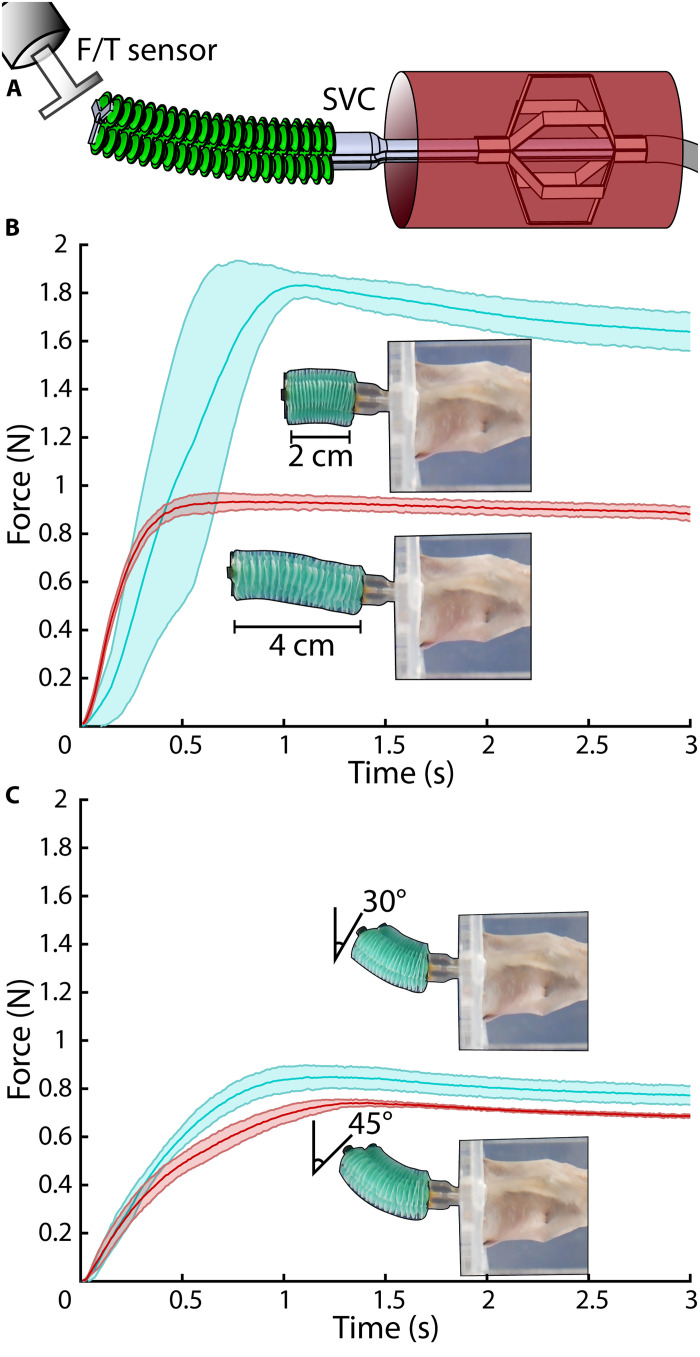
Integrated robotic platform ex vivo force generation. (**A**) The robot was deployed in a section of ex vivo porcine superior vena cava (SVC), and the soft manipulator component was pressed into a force sensor at various positions. (**B**) The manipulator’s force output was measured at 2 and 4 cm in extension. (**C**) The manipulator’s force output was measured at 30° and 45° in bending. In both bending cases, the target was positioned 3 cm from the manipulator’s base.

The device was deployed in a section of explanted porcine SVC to measure its force output in an ex vivo setting. Because puncture of the atrial septum, which is composed of tougher tissues than the TV annulus, can require forces of ≈1 N (*47*), we demonstrate that the integrated device can approach this requirement at multiple poses. Upon deployment of the stabilization, the soft manipulator maneuvered toward an acrylic target connected to a force sensor in four different orientations (Fig. 5A), pressing on the target until an internal pressure of 120 kPa caused the syringe motors to stall. The device generated a maximum force of 1.84 ± 0.05 N at 2-cm extension and 0.94 ± 0.03 N at 4-cm extension (Fig. 5B). It additionally generated a maximum force of 0.87 ± 0.05 N at 30° bending and 0.74 ± 0.01 N at 45° bending (Fig. 5C). In both bending cases, the target was placed 3 cm from the manipulator base to simulate the relative anatomical positions (*44*).

Once the manipulator made contact with the force sensor, the volume was maintained constant, and we observed a force decrease in all cases. In extension, the force decreased by 11.1% over 1.9 s at 20-mm extension and 6.3% over 2.4 s at 40-mm extension. In bending, the force decreased by 11.2% over 1.8 s at 30° bending and 8.1% over 1.8 s at 45° bending. This decrease is indicative of viscoelastic material behavior in the SVC sample (*67*). Additional details are provided in section S13.

#### In vitro demonstrations

**Fig. 6.**
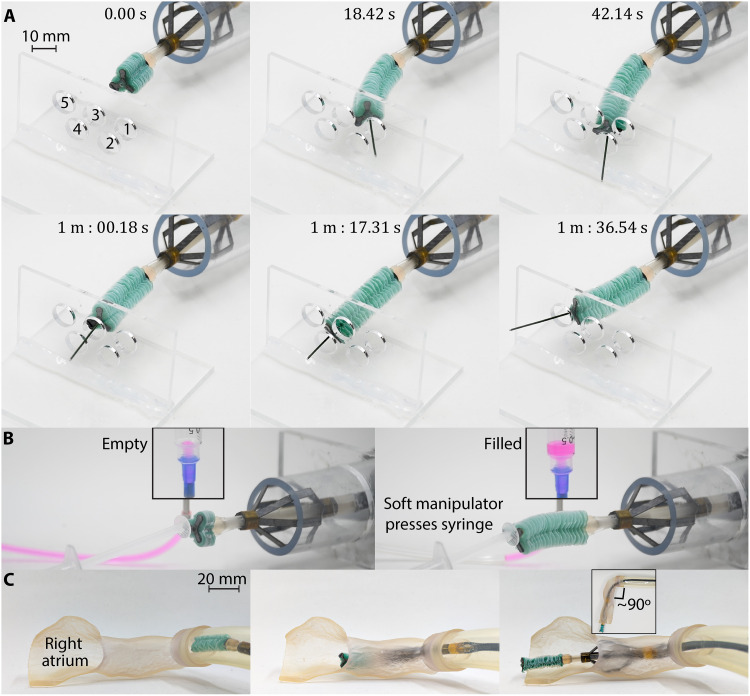
Robotic platform in vitro demonstrations. (**A**) The robot was deployed in a section of 25-mm tubing, and the soft manipulator guided a guidewire through five 1-cm holes in a timed trial. (**B**) Deployed in the same section of tubing, the soft manipulator extended to press on a syringe. The syringe was filled with dyed water and connected to a cylinder so that sufficient force generation from the integrated platform caused the cylinder to fill. (**C**) The robot is shown at different positions in a stereolithography-printed semiflexible right atrium (RA) model. The robot enters from the subclavian vein, which introduces a curvature of ≈90°.

The device was deployed in a section of clear 25-mm-diameter tubing to perform two demonstrations, highlighting its multifunctional abilities. First, the soft manipulator guided a 0.9-mm guidewire through five holes, each 1 cm in diameter, cut into a piece of acrylic and placed 4 cm from the base of the manipulator (Fig. 6A). The trial was timed and completed in just over 1 min and a half with the robot being operated by a single user. In addition to the dexterity demonstration, we also highlight the device’s force transmission. Using the same setup, this time the soft manipulator was actuated to press on a syringe plunger (Fig. 6B). The syringe was filled with dyed water and connected to a cylinder. By applying force on the plunger, the integrated device caused the cylinder to fill. When the stabilization mechanism was collapsed, the robotic platform was unable to successfully indent the syringe, instead sliding backward into the tubing as shown in movie S1. The robotic platform was further demonstrated to fit through peripheral vasculature such as the subclavian vein to access the RA (Fig. 6C). The device entered through a section of tubing and into an SLA-printed (Formlabs, Flexible 80A) semisoft RA model and was able to conform to the curved path. Figure 6C shows the robot entering the subclavian vein, traversing the ≈90° turn into the SVC, and bracing inside the entrance to the RA.

### Interventional validation

Ex vivo testing was performed to evaluate the device in the context of two exemplar procedures: CS lead placement and TV annular puncture. The goals of the ex vivo experimentation were: (i) to evaluate the stabilization mechanism within the SVC, (ii) to demonstrate the device’s ability to effectively perform in a fluidic environment, and (iii) to quantitatively assess our selected outcomes pertaining to CS cannulation and TV repair. The primary outcome was time to procedural completion. For CS cannulation, this was defined as the time required to navigate the soft manipulator toward the CS and cannulate the CS with a guidewire. For TV annular puncture, this was defined as the time required to navigate the soft manipulator toward the TV annulus, puncture the annulus, and retract. This represents a simplified version of a TV annuloplasty procedure, in which anchors are placed around the TV annulus and used to cinch it together with a secondary device. Because annuloplasty anchors and devices are proprietary and not standardized components, we instead demonstrate the ability to puncture the TV annulus at a predetermined location, which is paramount to the success of an annuloplasty procedure.

The secondary outcome for both CS cannulation and TV puncture was qualitative tissue damage at the SVC stabilization site via microscopic analysis. The TV puncture experiments had a tertiary outcome of contact force on a moving target.

#### Coronary sinus cannulation

CS lead placement is recommended as the initial method to treat certain types of heart failure (*71*). However, the catheters and guidewires used to conduct this procedure are often a source of difficulty given the RA’s challenging anatomy (*72*, *73*) and the limited dexterity of conventional instruments. Clinically, the time to CS cannulation with current techniques is often measured in hours rather than minutes. The main purpose of the CS cannulation task is to determine the ability of the soft robotic platform to navigate this difficult anatomy and complete the procedure.

**Fig. 7.**
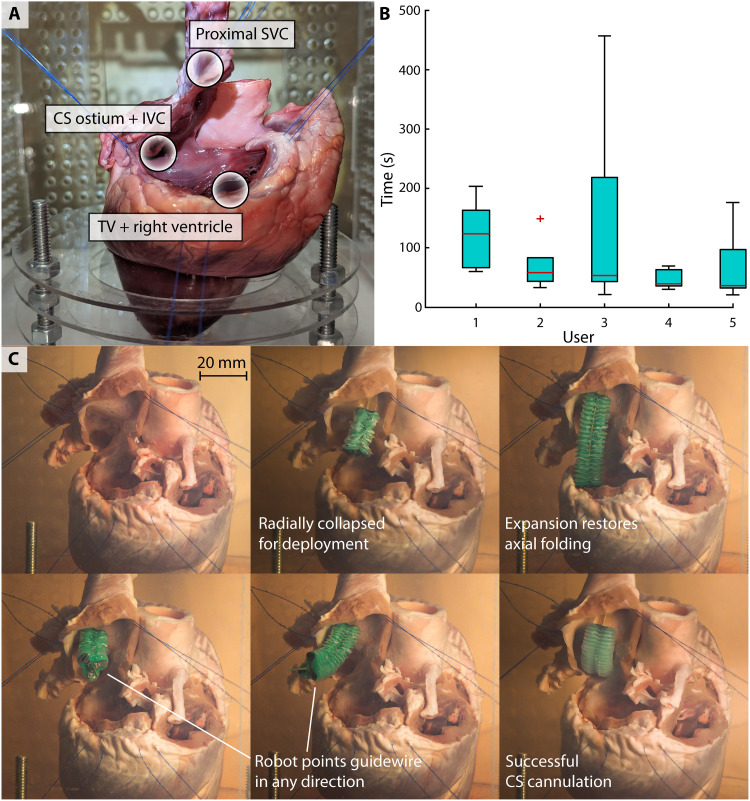
Coronary sinus (CS) cannulation task. (**A**) A porcine heart specimen was prepared to allow the user to directly visualize the superior vena cava (SVC) and CS. (**B**) Time to CS cannulation was measured for five users, each performing the procedure five times. (**C**) Representative stages in the CS cannulation procedure are depicted.

Five users deployed the device in an explanted porcine heart (Fig. 7A) and cannulated the CS five times each. Individual trials consisted of device insertion and stabilization in the SVC, device navigation toward the CS ostium, and guidewire cannulation of the CS. The operator began by inserting the device into the SVC until the catheter tip was observed partially extending into the RA. Ensuring a neutral, collapsed position of the soft manipulator before insertion helped avoid damage to the SVC tissue and to the device itself. After positioning the device, the stabilization mechanism was deployed. Subsequently, the soft manipulator was navigated under direct visualization toward the CS ostium. The tool guide’s central hole was oriented adjacent to and perpendicular with the CS ostium to allow for CS cannulation with a 0.9-mm guidewire.

The time to cannulation was recorded for each trial and reported with median, 25th and 75th percentile values, and range extremes (Fig. 7B). In the last two users, the median lies close to the 25th percentile values, indicating that a majority of trials were of shorter duration. The first two users exhibited a more evenly spread IQR despite the second user’s single statistical outlier. The overall mean time to cannulation across users after discarding each user’s slowest and fastest times was 68 ± 37 s. Representative steps of the procedure are shown in Fig. 7C and movie S2. In every test case, each user was able to approach procedural times on par with a control trial wherein an experienced clinician performed the task with an off-the-shelf curved-tip catheter. This comparison is demonstrated in movie S3.

#### Tricuspid valve annulus puncture

TV regurgitation is a result of various valve disease etiologies, and even mild to moderate cases present a notable risk to patients (*61*, *74*–*76*). A common approach to its treatment is annuloplasty, in which the annular tissue is cinched together with sutures. However, a minimally invasive approach to annuloplasty, which may involve the use of anchors to fasten an annuloplasty ring around the annulus, is challenging because of the valve’s motility and proximity to the atrioventricular node of the conduction pathway (*77*). These procedural issues make stable force transmission paramount to success. The main purpose of the TV annular puncture task is to simulate annuloplasty anchor placement by puncturing at a predetermined location without damaging surrounding structures such as the TV leaflets and applying consistent forces on a motile target. It was experimentally determined that 0.84 ± 0.12 N was sufficient to puncture the annular tissue (see section S8).

**Fig. 8.**
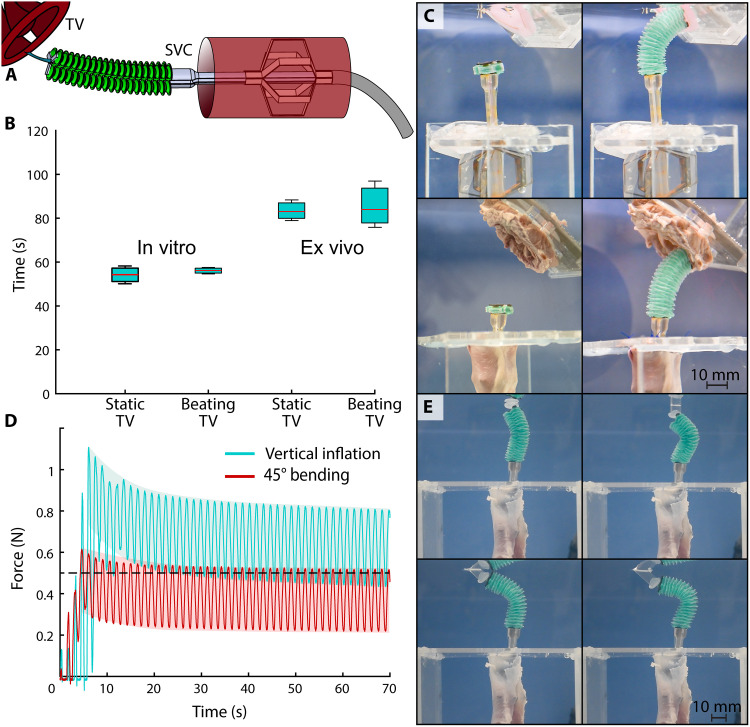
Tricuspid valve (TV) annular puncture task. (**A**) The device was deployed in both a TPU lumen and an explanted superior vena cava (SVC), and the soft manipulator guided a needle to puncture the TV annular tissue. The TV was positioned at 45° and 4 cm away relative to the base of the manipulator. (**B**) The time to complete a successful puncture was measured when the annulus was static and beating, for in vitro and ex vivo cases. (**C**) Representative images show successful puncture in both in vitro and ex vivo trials. (**D**) Force data were collected as the deployed robot made constant contact with a moving acrylic target in vertical and 45° configurations. (**E**) Representative images show the soft robot deforming with the target’s beating motions while still applying over 0.5 N of force.

The device was deployed in a section of explanted porcine SVC, and the TV annulus was punctured to simulate the robot’s ability to insert an annuloplasty anchor (Fig. 8A). Individual trials consisted of device insertion and stabilization in the SVC, device navigation toward the TV annulus, TV puncture, and dye injection shown in Fig. 8C and movie S4. The operator began by inserting the device into the SVC until the catheter tip was partially extended past the vein’s opening. Similar to the CS cannulation test, the device was inserted with the manipulator in a collapsed state, and the stabilization was deployed after device positioning. The soft manipulator then navigated toward the TV annulus, positioned 3 cm away and at a 45° angle to its base. The user delivered a needle through the tool guide to puncture the TV and deliver colored dye, indicating a successful puncture. This test was run in both an in vitro TV made of silicone (movie S4) and an explanted porcine TV (movie S5). Furthermore, in vitro and ex vivo trials were split into a static TV case and a beating TV case, in which a linear motor moved the TV to simulate cardiac pacing at 60 beats per minute (bpm) and a physiological amplitude of 6 mm oriented 45° from the longitudinal axis of the robot.

Different studies have measured different annular motion values depending on the reference plane (*78*, *79*), indicating its complex, multidimensional motion. Because the local base frame for the robot is in the relatively static SVC, these values do not necessarily reflect the motion from that reference point. Therefore, the motion parameters chosen for this test were deemed sufficient for preclinical operation on the TV. We also focused on simulating the ability of the robot to comply with the TV motion rather than pulsatile flow because the pressure and flow rate inside the RA are low compared to the left side of the heart. Mean pressures in the RA are approximately 8 mmHg (*66*). We validated experimentally that the contribution of pulsatile flow to device motion is negligible (see movie S8).

The median, 25th and 75th percentile values, and range extremes for procedural duration are reported (Fig. 8B). In the in vitro tests, the median time for the static case was 54.3 s, and the median time for the motile case was 56.2 s. In the ex vivo tests, the median time for the static case was 83.0 s, and the median time for the motile case was 84.0 s. From the static in vitro test to the beating-heart ex vivo test, the mean and median times to completion both increased, mirroring the increasing difficulty of each task (Fig. 8C).

In addition, the “beating-heart” contact trials demonstrate that the soft manipulator is capable of maintaining constant contact with the TV annulus by exploiting the inherent compliance of its constituent materials (Fig. 8D). We quantitatively determined that the device, when deployed in the ex vivo SVC, can maintain a contact force between 0.22 and 0.52 N after 70 s in 45° bending. When vertically inflated, the manipulator can maintain between 0.43 and 0.80 after 70 s. For both target locations, the robot successfully applied over half a newton of force at least once during each cycle, and it never lost contact until the end of the experiment (Fig. 8E). This corroborates the manipulator’s behavior shown in movies S4 to S6, where its inherent compliance allows it to move with the annular motion.

#### Robot deployment and removal

Separate from the two clinical tasks, the robot was also demonstrated during insertion to and removal from the heart. The robot was designed to fit through a 24-Fr introducer sheath. To fit, the soft manipulator must be radially collapsed, as shown in Fig. 3B and fig. S9A. Section S11 and movie S2 further detail the process of collapsing the soft manipulator, inserting the robot through the 24-Fr sheath for deployment, and radially collapsing again for removal through the sheath. Ultimately, the robot could be collapsed to a diameter of 8 mm for percutaneous cardiac access. Upon reaching the RA, the stabilization mechanism expanded to shape-lock against the SVC, while the soft manipulator expanded its nominal diameter to 15 mm to exploit the larger intracardiac workspace. The radial collapse process could also be performed remotely, with the robot inside the heart, to allow for removal through the same 24-Fr sheath. After removal, the robot remained intact and functional.

## DISCUSSION

We have introduced a soft robotic platform to address technical and clinical challenges in minimally invasive beating-heart intracardiac interventions. The platform is capable of collapsing to fit inside peripheral vascular access sites, shape-locking to stabilize against the proximal SVC and generate mechanical leverage, and guiding an interventional tool toward a target in the RA via a soft robotic manipulator. These features conform with both the vascular and intracardiac scales, provide a stable task space within the heart, and transmit forces on par with those required for tissue manipulation tasks. From a clinical perspective, the robot shows that by addressing all three key challenges of beating-heart intervention, a single platform can be applied to a range of procedures with inherently different tool requirements. Beyond the clinical perspective, this platform also opens a path toward soft robot–enabled direct manipulation tasks within the heart.

In vitro and ex vivo tests showed the critical role of the stabilization mechanism in enabling effective force transmission while minimizing physiological risks. The stabilization mechanism generated a negligible pressure gradient, on par with the baseline gradient from natural losses in pipe flow. Paired with the low stress concentrations imparted on the vasculature, the stabilization mechanism has the potential for extended deployment in the SVC and other large-diameter blood vessels.

Validation of the integrated robotic platform’s controllable, 3-DoF steering showed a measurable improvement in dexterity over commercially available catheters. In addition, by releasing the stabilization mechanism and reorienting the catheter, the operator can further expand the robot’s task space to reach a desired target. We also demonstrated the compatibility of the platform with conventional imaging methods such as x-ray fluoroscopy. The radiopaque materials used in the stabilization and tool guide components can be visualized under external imaging with a resolution that allows for the discrimination between each chamber of the soft robot (figs. S3 and S6). When deployed in an explanted section of SVC, the soft robot could apply forces on par with those required for tissue manipulation tasks. In the ex vivo CS cannulation test, the device enabled users to perform the task in times on par with an experienced clinician using conventional catheter instrumentation. Notably, four of five users had similar IQRs, regardless of their experience level. This demonstrates the platform’s accessibility and ease of use. In addition, the ex vivo TV annular puncture task shows that the robot can maintain constant contact for over a minute in a simulated beating heart environment. This highlights the device’s inherent stability without the need for expensive control hardware and imaging modalities and further highlights the feasibility of soft fluidic actuation in the beating heart environment. Last, the robot was able to collapse small enough to fit through a 24-Fr introducer sheath before expanding in the larger cardiac workspace, showing its ability to function in different length scales and compatibility with standard clinical instruments. In addition to addressing the size constraints of beating-heart interventions, the robot has demonstrated shape-locking and conformability for stability in a motile environment, and enough force generation to assist in transcatheter valve repair.

The preclinical ex vivo studies were designed to simulate a live animal test as closely as possible, but future in vivo tests will be necessary to move the technology toward clinical translation. During these live animal studies, we will perform a histological analysis on the intracardiac tissues that come into contact with the device, specifically the SVC. All adhesives and resins used in the fabrication of the robot are classified as biocompatible, and all clinical use cases for the robot last no longer than a few hours. However, histological analyses will verify whether material compatibility and stabilization stress cause excessive inflammation. Given that the site of stabilization in the proximal SVC is near the sinoatrial node of the conduction pathway, finding methods to limit inflammation will be crucial to avoid perioperative complications. The stabilization mechanism’s TPU coating layer is benign at a macroscopic level, but if excessive inflammation occurs in a live animal study, mitigation options include a hydrogel coating, which has been shown to have a high degree of compatibility with delicate tissue (*21*, *80*). In addition, potential x-ray fluoroscopy image attenuation due to extra layers of tissue may require investigation into more radiopaque materials, such as gold, to ensure clear visualization of the robot’s tip position. Furthermore, we demonstrated a computationally efficient kinematic model to approximate the robot’s pose. This method has the potential to address the inability of x-ray fluoroscopy to provide live robot position without exposure to harmful radiation. Integrating a model of the robot’s backbone with a static image of the anatomy generated by external imaging modalities would enable live navigation while reducing radiation exposure for the patient.

Ultimately, minimally invasive intracardiac intervention presents clinicians with a complex task owing to size constraints, workspace motion, and remote operation. By addressing all these major challenges in a single platform, this technology has the potential to open up a variety of minimally invasive beating-heart procedures to the general population, beyond just patients contraindicated for open surgery. In addition to existing procedures in interventional cardiology and EP, this technology is a step toward translating isolated TV repair from a risky operation to a more accessible interventional procedure. Increasing the accessibility of valve repair as an intervention rather than a surgery will ultimately provide a safer alternative to lower-risk individuals, enabling more frequent treatment.

The use of soft, accessible materials keeps overall costs low, which could minimize the financial impact on hospitals. The entire fabrication process relies on 2D and 3D additive manufacturing, and the actuation hardware can all fit on a single mayo tray stand (i.e., a small portable tray stand used to hold surgical instruments in the operating room) in a regular catheterization laboratory. The safety and accessibility of this soft robotic platform could enable its adoption in a variety of tissue manipulation tasks and make beating heart procedures more accessible to patients, clinicians, and hospitals. Last, given the disparity in equipment access between major hospitals and rural hospitals, this technology could provide a cost-effective method for rural doctors to perform life-saving procedures without needing to refer patients to a specialist that may be difficult to reach. In an effort to increase the accessibility of minimally invasive intracardiac therapies, we hope to tackle the health care inequality and therapeutic imbalances that contribute to the global burden of cardiovascular disease.

## MATERIALS AND METHODS

### Device fabrication

The stabilization mechanism was fabricated using layers of 250-μm 1095 spring steel, 24-μm polyimide (Kapton), and 180-μm TPU. The layers were individually cut on a 5-W UV laser and adhered with biocompatible pressure-activated adhesive (3M, 9877). Upon release from the sacrificial material, the laminate was wrapped around a 3D printed tube made of biocompatible resin (Formlabs, Biomed Amber). The actuating Bowden cable was constructed using Kevlar thread running through a polyethylene sheath (Braintree Scientific, PE 50). Two Bowden cables spaced 180° apart were found to distribute the actuating force more evenly. The soft manipulators were constructed using alternating layers of TPE and PTFE. These layers were individually cut on a CO_2_ laser, stacked and aligned, and bonded under 100 kPa at 140°C for a total duration of 40 min. The resulting laminate was released from its sacrificial material before 1-mm-diameter fluidic tubing (Braintree Scientific, RPT 40) was inserted into each chamber and adhered with biomedical Loctite adhesive (Loctite, 4861). The tool guide was fabricated using the same constituent layers as the stabilization mechanism, and the same laser cutting process. Upon release from its sacrificial material, the tool guide was mounted to the tip of the manipulator by adhering each of its three flexured lobes to one of the actuator chambers with biomedical Loctite adhesive.

The three fluidic pressure tubes, the two Bowden cable sheaths, and the central lumen (Duke Extrusion, Braided Stock Tubing with PTFE Liner) were all wrapped inside a 30-cm-long flexible braided tubing. On the user end of the device, the Bowden cable terminated in a two-way locking syringe (Fig. 3), allowing the user to quickly apply an actuating tension to the stabilization and lock its configuration. The fluidic tubing connected to custom volumetric control hardware, while the central lumen remained free to allow the user to insert and manipulate instruments.

### Data analysis

When analyzing data from device characterization tests, such as force or pressure tests, results are presented as the mean and SD across trials. When measuring the time to completion of clinical tasks, data are represented with median and IQR across trials. In the latter case, statistical outliers are represented in plots by the + symbol and calculated as *Q*1 − 1.5IQR or *Q*3 + 1.5IQR.

### Stabilization-induced pressure drop

The stabilization mechanism was inserted in a silicone tube flanked on either end by a pressure sensor (Nidec Copal Electronics Inc., P-7100-102G-M5). The sensors used have a maximum pressure of 100 kPa or 750 mmHg with a resolution of 0.2 kPa or 1.5 mmHg. The section of tubing was pressurized using a bucket filled with water at a depth of 10 cm to approximate the right heart flow pressure. Each sensor collected data for 100 s at a rate of 1000 Hz during the trials, allowing us to determine how the pressure gradient caused by each device behaved over time. The duration of 100 s was chosen to allow the flow and pressure values to stabilize.

### Stabilization-SVC interaction

The stabilization mechanism was fixed at one end, and a 50-N load cell (Instron, 5940) pulled on its Bowden cable to expand it. Another load cell (ATI Industrial Automation Inc., NANO17) placed next to the stabilization mechanism recorded the force generated by an individual linkage with respect to the Bowden cable’s tension. This test was then repeated with the stabilization free to expand inside a silicone lumen with an inner diameter of 25 mm.

The resulting force values were used as input for a finite element simulation in ABAQUS (Dassault Systemès) to model the interaction between the stabilization mechanism’s six linkages radially spaced along the SVC wall. The simulations had a total of 12,824 10-node quadratic tetrahedral elements between the SVC and stabilization mechanism linkages. The SVC was modeled as a linearly elastic material with properties based on the saphenous vein (*68*) because of the lack of hyperelastic SVC models available in the literature. It was modeled with an inner diameter of 25 mm and a thickness of 2 mm, and a gradient seed size from 1 to 5 mm was applied. The individual linkages were modeled according to the parameters of their constituent materials. For the TPU coat, we used a Young’s modulus of 12 MPa, a Poisson’s ratio of 0.5, and a density of 1082 kg/m^3^ (*81*). The linkages were approximated as stainless steel with Young’s modulus of 205 GPa, a Poisson’s ratio of 0.29, and a density of 7750 kg/m^3^. Ten-node quadratic tetrahedron elements were used with a gradient seed size from 1 to 2 mm for the TPU elements and a universal seed size of 2 mm for the steel elements. Each linkage was 2.9 mm wide and 15 mm long. The steel layer had a thickness of 0.8 mm, while the TPU coating layer had a curved surface with minimum thickness of 0.10 mm and maximum thickness of 0.18 mm.

### Axial and radial force

The stabilization mechanism was deployed in an explanted section of porcine SVC, as well as a lumen made of TPU. In both cases, an acrylic indenter attached to a load cell (ATI Industrial Automation, Nano17) mounted on a robot arm (Universal Robots) pressed on the tip of the stabilization mechanism in the axial and radial directions *n* = 5 times each.

### Soft robot workspace

A 3D model of the RA was generated by segmenting a CT scan of a heart in Mimics Innovation Suite (Materialise). The resulting STL mesh represents the internal geometry of the segmented anatomy. Separately, the soft manipulator was actuated to demonstrate the extremes of its workspace using three syringe pumps (Harvard Apparatus), one for each fluidic chamber. A Cartesian product of five volumetric increments from 0 to 5 ml in each fluidic chamber determined the volumetric actuation states. The syringe pumps were programmed to automatically sweep through this list of volumetric inputs. At each corresponding state, an electromagnetic position tracker (Northern Digital Inc., Aurora) recorded the tip position in 3D Euclidean space. The resulting point cloud was overlaid inside the RA mesh such that the base of the manipulator was positioned at the opening of the SVC.

### Full robot blocked force

The device was deployed in a section of explanted porcine SVC placed underwater. An acrylic indenter attached to a load cell (ATI Industrial Automation, Nano17) mounted on a robot arm (Universal Robots) was positioned in four different locations relative to the manipulator’s base: 2 and 4 cm away in extension and 3 cm away at a 30° and 45° angle. The manipulator was then remotely controlled to push against the indenter in each of these positions while the force sensor recorded the resulting force output. In all cases, mean and SD were calculated over *n* = 5 trials, except for the 30° bending case, in which one trial was discarded because of the manipulator sliding with respect to the acrylic target.

### Clinical intervention

Porcine adult hearts (450 to 500 g) were acquired from LAMPIRE Biological Laboratories (Pipersville, PA) with a special request for preserved SVC and stored at −80°C when not in use to preserve tissue integrity. The RA was incised anteromedially with a #11 blade superior to the atrioventricular groove to avoid the TV annulus. This incisional plane was extended medially to the septal wall using Metzenbaum scissors. The dissection was then directed superiorly onto the RA appendage (RAA). A majority of the RAA was resected, ensuring a sufficient tissue cuff around the aorta and SVC to provide locations for subsequent stay suture placement. The resection was carried inferiorly along the lateral border of the RA following the terminal sulcus. Care was taken to avoid disruption of the SVC or inferior vena cava (IVC). Last, the dissection was completed inferiorly with connection to the initial incision site and removal of the RA free wall. As a result, the entire RA free wall was resected, leaving the entirety of the TV annulus, CS ostium, SVC, and IVC intact.

#### CS cannulation

The heart was suspended in an acrylic support structure to allow for aquatic device testing. Five to six 3-0 Prolene horizontal mattress stay sutures were placed in attempts to reestablish normal anatomic/geometric relationships to increase the validity of the ex vivo model. The SVC was directed superiorly to accommodate device insertion, and appropriate tension was applied to prevent distortion of the heart and kinking of the IVC and CS ostium. The heart and acrylic support structure were then submerged in a water bath.

Each operator performed *n* = 5 recorded device experimental test runs to increase the reliability of the collected quantitative data. Each run consisted of device insertion and stabilization in the SVC, device navigation to the CS ostia, and catheter cannulation of the CS. The operator began by inserting the device into the SVC until the catheter tip was observed partially extending into the RA. After appropriate positioning was verified, the stabilization mechanism was deployed. Subsequently, the soft manipulator was navigated under direct visualization toward the CS ostium via remote control. Ultimately, the tool guide was positioned adjacent to the CS ostium to allow for intubation of the CS with a guidewire.

After an experienced clinician verified guidewire insertion, the time required to complete the test run was recorded. Furthermore, the SVC was harvested for qualitative microscopic tissue analysis. The resulting image is composed by stitching together 12 constituent images and generating a blank, black background around the tissue sample. Original images are shown in the Supplementary Materials.

#### TV annular puncture

The heart was further dissected to retrieve a 5-cm-long section of the SVC, as well as the TV, leaflets, and annular tissue. The device was tested in both in vitro and ex vivo settings. In the in vitro case, the device was deployed in a lumen made of TPU. It was then remotely controlled to move toward a TV annulus molded from silicone (Ecoflex 30). Both the SVC and TV annulus were sutured to acrylic fixtures to maintain their shape during device operation. Upon the manipulator making contact with the annulus, a needle was pushed through the device’s central lumen to puncture the target. Dye-colored water was then injected through the needle to indicate a successful puncture. The ex vivo case was run in an identical manner to the in vitro case but with the dissected section of porcine SVC and the explanted porcine TV annulus. In addition, the TV annulus samples were attached to a linear motor (Actuonix, S20), allowing them to programmatically beat at 60 bpm and an amplitude of 6 mm per cycle. The in vitro and ex vivo tests, in both static and beating cases, were performed *n* = 3 times each to increase the validity of the resulting procedural durations.

Upon completion of the annular puncture experiments, the TV annulus was replaced with an acrylic target, which was programmed to beat with the same amplitude as the TV annulus but at 40 bpm. The slower frequency was set to better visualize the force generated by the device on the beating target. The soft manipulator was directed toward the target and maintained contact for ≈60 s, yielding an oscillating force output.

## Supplementary Materials

### This PDF file includes:

Sections S1 to S13 Figs. S1 to S11 Table S1 Legends for movies S1 to S9 References

## References

[1] P. E. Dupont, B. J. Nelson, M. Goldfarb, B. Hannaford, A. Menciassi, M. K. O’Malley, N. Simaan, P. Valdastri, G. Z. Yang, A decade retrospective of medical robotics research from 2010 to 2020. Sci. Robot. 6, eabi8017 (2021). 34757801 10.1126/scirobotics.abi8017 PMC8890492

[2] C. Freschi, V. Ferrari, F. Melfi, M. Ferrari, F. Mosca, A. Cuschieri, Technical review of the da vinci surgical telemanipulator. Int. J. Med. Robot. 9, 396–406 (2013). 23166047 10.1002/rcs.1468

[3] L. Nagaraju, D. Menon, P. F. Aziz, Use of 3d electroanatomical navigation (carto-3) to minimize or eliminate fluoroscopy use in the ablation of pediatric supraventricular tachyarrhythmias. Pacing Clin. Electrophysiol. 39, 574–580 (2016). 26873564 10.1111/pace.12830

[4] M. Giaccardi, G. Mascia, A. P. Perini, A. Giomi, S. Cartei, M. Milli, Long-term outcomes after zero x-ray arrhythmia ablation. J. Interv. Card. Electrophysiol. 54, 43–48 (2019). 29948584 10.1007/s10840-018-0390-7

[5] R. Tarwala, L. D. Dorr, Robotic assisted total hip arthroplasty using the MAKO platform. Curr. Rev. Musculoskelet. Med. 4, 151–156 (2011). 21728013 10.1007/s12178-011-9086-7 PMC3261258

[6] G. Fagogenis, M. Mencattelli, Z. Machaidze, B. Rosa, K. Price, F. Wu, V. Weixler, M. Saeed, J. E. Mayer, P. E. Dupont, Autonomous robotic intracardiac catheter navigation using haptic vision. Sci. Robot. 4, eaaw1977 (2019). 31414071 10.1126/scirobotics.aaw1977 PMC6693882

[7] A. Ataollahi, I. Berra, N. V. Vasilyev, Z. Machaidze, P. E. Dupont, Cardioscopic tool-delivery instrument for beating-heart surgery. IEEE/ASME Trans. Mechatron. 21, 584–590 (2016). 26951754 10.1109/TMECH.2015.2494842 PMC4778079

[8] N. V. Vasilyev, A. H. Gosline, A. Veeramani, M. T. Wu, G. P. Schmitz, R. T. Chen, V. Arabagi, P. J. D. Nido, P. E. Dupont, Tissue removal inside the beating heart using a robotically delivered metal mems tool. Int. J. Robot. Res. 34, 236–247 (2015). 10.1177/0278364914543671 PMC13278507 42327924

[9] X. Zhang, M. C. Palumbo, F. Perico, M. Magro, A. Fortuna, T. Magni, E. Votta, A. Segato, E. D. Momi, Robotic actuation and control of a catheter for structural intervention cardiology, in *IEEE International Conference on Intelligent Robots and Systems (IROS)* (IEEE, 2022), pp. 5907–5913 (2022).

[10] P. M. Loschak, A. Degirmenci, C. M. Tschabrunn, E. Anter, R. D. Howe, Automatically steering cardiac catheters in vivo with respiratory motion compensation. Int. J. Robot. Res. 39, 586–597 (2020). 10.1177/0278364920903785 PMC7357965 32661450

[11] R. Qi, N. U. Nayar, J. P. Desai, Telerobotic transcatheter delivery system for mitral valve implant. IEEE Robot. Autom. Lett., 1–8 (2023). 10.1109/LRA.2023.3265592 PMC10751040 38152328

[12] M. Rox, D. S. Esser, M. E. Smith, T. E. Ertop, M. Emerson, F. Maldonado, E. A. Gillaspie, A. Kuntz, R. J. Webster, Toward continuum robot tentacles for lung interventions: Exploring folding support disks. IEEE Robot. Autom. Lett. 8, 3494–3501 (2023). 37333046 10.1109/LRA.2023.3267006 PMC10270676

[13] Y. Kim, S. S. Cheng, M. Diakite, R. P. Gullapalli, J. M. Simard, J. P. Desai, Toward the development of a flexible mesoscale mri-compatible neurosurgical continuum robot. IEEE Transact. Robot. 33, 1386–1397 (2017). 10.1109/TRO.2017.2719035 PMC5718214 29225557

[14] J. W. Martin, L. Barducci, B. Scaglioni, J. C. Norton, C. Winters, V. Subramanian, A. Arezzo, K. L. Obstein, P. Valdastri, Robotic autonomy for magnetic endoscope biopsy. IEEE Trans. Med. Robot. Bionics 4, 599–607 (2022). 36249558 10.1109/TMRB.2022.3187028 PMC9555223

[15] M. Cianchetti, T. Ranzani, G. Gerboni, T. Nanayakkara, K. Althoefer, P. Dasgupta, A. Menciassi, Soft robotics technologies to address shortcomings in today’s minimally invasive surgery: The stiff-flop approach. Soft Robotics 1, 122–131 (2014).

[16] M. Cianchetti, C. Laschi, A. Menciassi, P. Dario, Biomedical applications of soft robotics. Nat. Rev. Mater. 3, 143–153 (2018).

[17] M. Runciman, A. Darzi, G. P. Mylonas, Soft robotics in minimally invasive surgery. Soft Robot. 6, 423–443 (2019). 30920355 10.1089/soro.2018.0136 PMC6690729

[18] K. Ikuta, Y. Matsuda, D. Yajima, Y. Ota, Pressure pulse drive: A control method for the precise bending of hydraulic active catheters. IEEE/ASME Transact. Mechatron. 17, 876–883 (2012).

[19] M. Li, R. Obregon, J. J. Heit, A. Norbash, E. W. Hawkes, T. K. Morimoto, Vine catheter for endovascular surgery. IEEE Trans. Med. Robot. Bionics 3, 384–391 (2021).

[20] C. Fischer, Q. Boehler, B. J. Nelson, Using magnetic fields to navigate and simultaneously localize catheters in endoluminal environments. IEEE Robot. Autom. Lett. 7, 7217–7223 (2022).

[21] Y. Kim, G. A. Parada, S. Liu, X. Zhao, Ferromagnetic soft continuum robots. Sci. Robot. 4, eaax7329 (2019). 33137788 10.1126/scirobotics.aax7329

[22] T. L. Thomas, J. Sikorski, G. K. Ananthasuresh, V. K. Venkiteswaran, S. Misra, Design, sensing, and control of a magnetic compliant continuum manipulator. IEEE Trans. Med. Robot. Bionics 4, 910–921 (2022).

[23] E. T. Roche, M. A. Horvath, I. Wamala, A. Alazmani, S. E. Song, W. Whyte, Z. Machaidze, C. J. Payne, J. C. Weaver, G. Fishbein, J. Kuebler, N. V. Vasilyev, D. J. Mooney, F. A. Pigula, C. J. Walsh, Soft robotic sleeve supports heart function. Sci. Transl. Med. 9, eaaf3925 (2017). 28100834 10.1126/scitranslmed.aaf3925

[24] C. J. Payne, I. Wamala, D. Bautista-Salinas, M. Saeed, D. V. Story, T. Thalhofer, M. A. Horvath, C. Abah, P. J. D. Nido, C. J. Walsh, N. V. Vasilyev, Soft robotic ventricular assist device with septal bracing for therapy of heart failure. Sci. Robot. 2, eaan6736 (2017). 33157903 10.1126/scirobotics.aan6736

[25] N. A. Patronik, C. N. Riviere, S. E. Qarra, M. A. Zenati, The heartlander: A novel epicardial crawling robot for myocardial injections. Int. Congr. Ser. 1281, 735–739 (2005).

[26] N. A. Patronik, M. A. Zenati, C. N. Riviere, C. N. Riviere, Preliminary evaluation of a mobile robotic device for navigation and intervention on the beating heart. Comput. Aided Surg. 10, 225–232 (2010). 10.3109/10929080500230197 16393791

[27] Y. Kim, E. Genevriere, P. Harker, J. Choe, M. Balicki, R. W. Regenhardt, J. E. Vranic, A. A. Dmytriw, A. B. Patel, X. Zhao, Telerobotic neurovascular interventions with magnetic manipulation. Sci. Robot. 7, eabg9907 (2022). 35417201 10.1126/scirobotics.abg9907 PMC9254892

[28] T. Amadeo, D. V. Lewen, T. Janke, T. Ranzani, A. Devaiah, U. Upadhyay, S. Russo, Soft robotic deployable origami actuators for neurosurgical brain retraction. Front. Robot. AI 8, 731010 (2022). 35096979 10.3389/frobt.2021.731010 PMC8795889

[29] D. Van Lewen, T. Janke, H. Lee, R. Austin, E. Billatos, S. Russo, A millimeter-scale soft robot for tissue biopsy procedures. Adv. Intell. Syst. 5, 2200326 (2023). 37637939 10.1002/aisy.202200326 PMC10456987

[30] M. McCandless, A. Perry, N. DiFilippo, A. Carroll, E. Billatos, S. Russo, A soft robot for peripheral lung cancer diagnosis and therapy. Soft Robot. 9, 754–766 (2021). 34357810 10.1089/soro.2020.0127

[31] G. Pittiglio, P. Lloyd, T. D. Veiga, O. Onaizah, C. Pompili, J. H. Chandler, P. Valdastri, Patient-specific magnetic catheters for atraumatic autonomous endoscopy. Soft Robot. 9, 1120–1133 (2022). 35312350 10.1089/soro.2021.0090 PMC9805888

[32] M. McCandless, A. Gerald, A. Carroll, H. Aihara, S. Russo, A soft robotic sleeve for safer colonoscopy procedures. IEEE Robot. Autom. Lett. 6, 5292–5299 (2021). 34027062 10.1109/lra.2021.3073651 PMC8132950

[33] D. Son, H. Gilbert, M. Sitti, Magnetically actuated soft capsule endoscope for fine-needle biopsy. Soft Robot. 7, 10–21 (2020). 31418640 10.1089/soro.2018.0171

[34] S. Russo, T. Ranzani, C. J. Walsh, R. J. Wood, An additive millimeter-scale fabrication method for soft biocompatible actuators and sensors. Adv. Mater. Technol. 2, 1700135 (2017).

[35] T. Ranzani, G. Gerboni, M. Cianchetti, A. Menciassi, A bioinspired soft manipulator for minimally invasive surgery. Bioinspir. Biomim. 10, 035008 (2015). 25970550 10.1088/1748-3190/10/3/035008

[36] C. W. Tsao, A. W. Aday, Z. I. Almarzooq, A. Alonso, A. Z. Beaton, M. S. Bittencourt, A. K. Boehme, A. E. Buxton, A. P. Carson, Y. Commodore-Mensah, M. S. Elkind, K. R. Evenson, C. Eze-Nliam, J. F. Ferguson, G. Generoso, J. E. Ho, R. Kalani, S. S. Khan, B. M. Kissela, K. L. Knutson, D. A. Levine, T. T. Lewis, J. Liu, M. S. Loop, J. Ma, M. E. Mussolino, S. D. Navaneethan, A. M. Perak, R. Poudel, M. Rezk-Hanna, G. A. Roth, E. B. Schroeder, S. H. Shah, E. L. Thacker, L. B. Vanwagner, S. S. Virani, J. H. Voecks, N. Y. Wang, K. Yaffe, S. S. Martin, Heart disease and stroke statistics–2022 update: A report from the american heart association. Circulation 145, E153–E639 (2022). 35078371 10.1161/CIR.0000000000001052

[37] S. Coffey, R. Roberts-Thomson, A. Brown, J. Carapetis, M. Chen, M. Enriquez-Sarano, L. Zühlke, B. D. Prendergast, Global epidemiology of valvular heart disease. Nat. Rev. Cardiol. 18, 853–864 (2021). 34172950 10.1038/s41569-021-00570-z

[38] Y. Wang, R. Bellomo, Cardiac surgery-associated acute kidney injury: Risk factors, pathophysiology and treatment. Nat. Rev. Nephrol. 13, 697–711 (2017). 28869251 10.1038/nrneph.2017.119

[39] J. Burgner-Kahrs, D. C. Rucker, H. Choset, Continuum robots for medical applications: A survey. IEEE Trans. Robot. 31, 1261–1280 (2015).

[40] J. Rodés-Cabau, R. T. Hahn, A. Latib, M. Laule, A. Lauten, F. Maisano, J. Schofer, F. Campelo-Parada, R. Puri, A. Vahanian, Transcatheter therapies for treating tricuspid regurgitation. J. Am. Coll. Cardiol. 67, 1829–1845 (2016). 27081024 10.1016/j.jacc.2016.01.063

[41] A. Lauten, H. R. Figulla, A. Unbehaun, N. Fam, J. Schofer, T. Doenst, J. Hausleiter, M. Franz, C. Jung, H. Dreger, D. Leistner, B. Alushi, A. Stundl, U. Landmesser, V. Falk, K. Stangl, M. Laule, Interventional treatment of severe tricuspid regurgitation. Circ. Cardiovasc. Interv. 11, e006061 (2018). 29445001 10.1161/CIRCINTERVENTIONS.117.006061

[42] L. Asmarats, R. Puri, A. Latib, J. L. Navia, J. Rodés-Cabau, Transcatheter tricuspid valve interventions: Landscape, challenges, and future directions. J. Am. Coll. Cardiol. 71, 2935–2956 (2018). 29929618 10.1016/j.jacc.2018.04.031

[43] K. Matli, A. Mahdi, V. Zibara, C. Costanian, G. Ghanem, Transcatheter tricuspid valve intervention techniques and procedural steps for the treatment of tricuspid regurgitation: a review of the literature. Open Heart 9, e002030 (2022). 35654481 10.1136/openhrt-2022-002030 PMC9163538

[44] K. Keller, C. Sinning, A. Schulz, C. Jünger, V. H. Schmitt, O. Hahad, T. Zeller, M. Beutel, N. Pfeiffer, K. Strauch, S. Blankenberg, K. J. Lackner, J. H. Prochaska, E. Schulz, T. Münzel, P. S. Wild, Right atrium size in the general population. Sci. Rep. 11, 22523 (2021). 34795353 10.1038/s41598-021-01968-y PMC8602329

[45] E. S. Gang, B. L. Nguyen, Y. Shachar, L. Farkas, L. Farkas, B. Marx, D. Johnson, M. C. Fishbein, C. Gaudio, S. J. Kim, Dynamically shaped magnetic fields: Initial animal validation of a new remote electrophysiology catheter guidance and control system. Circ. Arrhythm. Electrophysiol. 4, 770–777 (2011). 21690463 10.1161/CIRCEP.110.959692

[46] S. Kumar, J. B. Morton, K. Halloran, S. J. Spence, G. Lee, M. C. Wong, P. M. Kistler, J. M. Kalman, Effect of respiration on catheter-tissue contact force during ablation of atrial arrhythmias. Heart Rhythm 9, 1041–1047.e1 (2012). 22342855 10.1016/j.hrthm.2012.02.015

[47] C. R. Wagner, D. P. Perrin, R. D. Howe, N. Vasilyev, P. J. D. Nido, Force feedback in a three-dimensional ultrasound-guided surgical task, in *14th Symposium on Haptics Interfaces for Virtual Environment and Teleoperator Systems (HAPTICS 2006)* (IEEE, 2006), pp. 43–48

[48] S. B. Kesner, R. D. Howe, Robotic catheter cardiac ablation combining ultrasound guidance and force control. Int. J. Robot. Res. 33, 631–644 (2014).

[49] N. V. Vasilyev, A. H. Gosline, E. Butler, N. Lang, P. J. Codd, H. Yamauchi, E. N. Feins, C. R. Folk, A. L. Cohen, R. Chen, D. Zurakowski, P. J. D. Nido, P. E. Dupont, Percutaneous steerable robotic tool delivery platform and metal microelectromechanical systems device for tissue manipulation and approximation: Closure of patent foramen ovale in an animal model. Circ. Cardiovasc. Interv. 6, 468–475 (2013). 23899870 10.1161/CIRCINTERVENTIONS.112.000324 PMC3837556

[50] L. Cruddas, G. Martin, C. Riga, Robotic endovascular surgery: Current and future practice. Semin. Vasc. Surg. 34, 233–240 (2021). 34911629 10.1053/j.semvascsurg.2021.10.002

[51] A. Püschel, C. Schafmayer, J. Groß, Robot-assisted techniques in vascular and endovascular surgery. Langenbecks Arch. Surg. 407, 1789–1795 (2022). 35226179 10.1007/s00423-022-02465-0 PMC8884093

[52] M. Mhanna, A. Beran, A. Al-Abdouh, O. Sajdeya, M. Barbarawi, M. Alsaiqali, A. Jabri, A. Al-Aaraj, A. Alharbi, P. Chacko, Steerable versus nonsteerable sheath technology in atrial fibrillation ablation: A systematic review and meta-analysis. J. Arrhythm. 38, 570–579 (2022). 35936032 10.1002/joa3.12742 PMC9347204

[53] M. Singh, C. E. Varela, W. Whyte, M. A. Horvath, N. C. S. Tan, C. B. Ong, P. Liang, M. L. Schermerhorn, E. T. Roche, T. W. J. Steele, Minimally invasive electroceutical catheter for endoluminal defect sealing. Sci. Adv. 7, eabf6855 (2021). 33811080 10.1126/sciadv.abf6855 PMC11057783

[54] E. T. Roche, A. Fabozzo, Y. Lee, P. Polygerinos, I. Friehs, L. Schuster, W. Whyte, A. M. C. Berazaluce, A. Bueno, N. Lang, M. J. N. Pereira, E. Feins, S. Wasserman, E. D. O’Cearbhaill, N. V. Vasilyev, D. J. Mooney, J. M. Karp, P. J. del Nido, C. J. Walsh, A light-reflecting balloon catheter for atraumatic tissue defect repair. Sci. Transl. Med. 7, 306ra149 (2015). 10.1126/scitranslmed.aaa2406 26400910

[55] A. Ali, D. Dodou, G. Smit, R. Rink, P. Breedveld, Stabilizing interventional instruments in the cardiovascular system: A classification of mechanisms. Med. Eng. Phys. 89, 22–32 (2021). 33608122 10.1016/j.medengphy.2021.01.004

[56] J. J. Zhou, A. Quadri, A. Sewani, Y. Alawneh, R. Gilliland-Rocque, C. Magnin, A. Dueck, G. A. Wright, M. A. Tavallaei, The cathpilot: A novel approach for accurate interventional device steering and tracking. IEEE/ASME Trans. Mechatron. 27, 5812–5823 (2022).

[57] L. P. Gaffney, P. M. Loschak, R. D. Howe, A deployable transseptal brace for stabilizing cardiac catheters. J. Mech. Des. N Y 140, 0750031–7500312 (2018). 30083041 10.1115/1.4039495 PMC6056188

[58] S. B. Kesner, R. D. Howe, Position control of motion compensation cardiac catheters. IEEE Trans. Robot. 27, 1045–1055 (2011). 10.1109/TRO.2011.2160467 PMC3160644 21874124

[59] A. Degirmenci, P. M. Loschak, C. M. Tschabrunn, E. Anter, R. D. Howe, Compensation for unconstrained catheter shaft motion in cardiac catheters. IEEE Int. Conf. Robot. Autom. 2016, 4436–4442 (2016). 27525170 10.1109/ICRA.2016.7487643 PMC4980095

[60] N. U. Nayar, R. Qi, J. P. Desai, Toward the design and development of a robotic transcatheter delivery system for mitral valve implant. IEEE Trans. Med. Robot. Bionics 4, 922–934 (2022). 37214350 10.1109/tmrb.2022.3215522 PMC10198124

[61] L. Gheorghe, B. J. Rensing, J. A. V. der Heyden, B. Rana, M. C. Post, M. J. Swaans, Tricuspid regurgitation: No longer the ‘forgotten’ valve. EMJ Cardiol. 7, 119–127 (2019).

[62] A. Parolari, F. Barili, A. Pilozzi, D. Pacini, Ring or suture annuloplasty for tricuspid regurgitation? A meta-analysis review. Ann. Thorac. Surg. 98, 2255–2263 (2014). 25443026 10.1016/j.athoracsur.2014.06.100

[63] J. Gafford, T. Ranzani, S. Russo, A. Degirmenci, S. Kesner, R. Howe, R. Wood, C. Walsh, Toward medical devices with integrated mechanisms, sensors, and actuators via printed-circuit mems. J. Med. Devices 11, 011007 (2017).

[64] S. Becker, T. Ranzani, S. Russo, R. J. Wood, Pop-up tissue retraction mechanism for endoscopic surgery, in *IEEE International Conference on Intelligent Robots and Systems* (IEEE, 2017), pp, 920–927.

[65] T. Ranzani, S. Russo, N. W. Bartlett, M. Wehner, R. J. Wood, Increasing the dimensionality of soft microstructures through injection-induced self-folding. Adv. Mater. 30, 1–15 (2018). 10.1002/adma.201802739 30079470

[66] R. Beigel, B. Cercek, H. Luo, R. J. Siegel, Noninvasive evaluation of right atrial pressure. J. Am. Soc. Echocardiogr. 26, 1033–1042 (2013). 23860098 10.1016/j.echo.2013.06.004

[67] D. B. Camasão, D. Mantovani, The mechanical characterization of blood vessels and their substitutes in the continuous quest for physiological-relevant performances. a critical review. Mater. Today Bio 10, 100106 (2021). 10.1016/j.mtbio.2021.100106 PMC8050780 33889837

[68] R. J. Gusic, M. Petko, R. Myung, J. W. Gaynor, K. J. Gooch, Mechanical properties of native and ex vivo remodeled porcine saphenous veins. J. Biomech. 38, 1770–1779 (2005). 15936764 10.1016/j.jbiomech.2005.04.002

[69] J. Rogatinsky, K. Gomatam, Z. H. Lim, M. Lee, L. Kinnicutt, C. Duriez, P. Thomson, K. McDonald, T. Ranzani, A collapsible soft actuator facilitates performance in constrained environments. Adv. Intell. Syst. 4, 2200085 (2022). 37449010 10.1002/aisy.202200085 PMC10338025

[70] T. Ranzani, S. Russo, F. Schwab, C. J. Walsh, R. J. Wood, Deployable stabilization mechanisms for endoscopic procedures, in *Proceedings - IEEE International Conference on Robotics and Automation* (ICRA, 2017), pp. 1125–1131.

[71] A. S. Tang, G. A. Wells, M. Talajic, M. O. Arnold, R. Sheldon, S. Connolly, S. H. Hohnloser, G. Nichol, D. H. Birnie, J. L. Sapp, R. Yee, J. S. Healey, J. L. Rouleau, Cardiac-resynchronization therapy for mild-to-moderate heart failure. N. Engl. J. Med. 363, 2385–2395 (2010). 21073365 10.1056/NEJMoa1009540

[72] A. D. Costa, A. Gate-Martinet, P. Rouffiange, A. Cerisier, A. Nadrouss, L. Bisch, C. Romeyer-Bouchard, K. Isaaz, Anatomical factors involved in difficult cardiac resynchronization therapy procedure: A non-invasive study using dual-source 64-multi-slice computed tomography. Europace 14, 833–840 (2012). 22117034 10.1093/europace/eur350

[73] A. Noheria, C. V. Desimone, N. Lachman, W. D. Edwards, A. S. Gami, J. J. Maleszewski, P. A. Friedman, T. M. Munger, S. C. Hammill, D. L. Hayes, D. L. Packer, S. J. Asirvatham, Anatomy of the coronary sinus and epicardial coronary venous system in 620 hearts: An electrophysiology perspective. J. Cardiovasc. Electrophysiol. 24, 1–6 (2013). 23066703 10.1111/j.1540-8167.2012.02443.x

[74] G. Benfari, C. Antoine, W. L. Miller, P. Thapa, Y. Topilsky, A. Rossi, H. I. Michelena, S. Pislaru, M. Enriquez-Sarano, Excess mortality associated with functional tricuspid regurgitation complicating heart failure with reduced ejection fraction. Circulation 140, 196–206 (2019). 31117814 10.1161/CIRCULATIONAHA.118.038946

[75] E. Chorin, Z. Rozenbaum, Y. Topilsky, M. Konigstein, T. Ziv-Baran, E. Richert, G. Keren, S. Banai, Tricuspid regurgitation and long-term clinical outcomes. Eur. Heart J. Cardiovasc. Imaging 21, 157–165 (2019). 10.1093/ehjci/jez216 31544933

[76] D. Messika-Zeitoun, P. Verta, J. Gregson, S. J. Pocock, I. Boero, T. E. Feldman, W. T. Abraham, J. A. Lindenfeld, J. Bax, M. Leon, M. Enriquez-Sarano, Impact of tricuspid regurgitation on survival in patients with heart failure: A large electronic health record patient-level database analysis. Eur. J. Heart Fail. 22, 1803–1813 (2020). 32367642 10.1002/ejhf.1830

[77] S. Singh-Gryzbon, A. W. Siefert, E. L. Pierce, A. P. Yoganathan, Tricuspid valve annular mechanics: Interactions with and implications for transcatheter devices. Cardiovasc. Eng. Technol. 10, 193–204 (2019). 30756336 10.1007/s13239-019-00405-6

[78] K. Owais, C. E. Taylor, L. Jiang, K. R. Khabbaz, M. Montealegre-Gallegos, R. Matyal, J. H. Gorman, R. C. Gorman, F. Mahmood, Tricuspid annulus: A three-dimensional deconstruction and reconstruction. Ann. Thorac. Surg. 98, 1536–1542 (2014). 25249160 10.1016/j.athoracsur.2014.07.005 PMC6563329

[79] K. Emilsson, R. Egerlid, B. Nygren, Tricuspid annulus motion and mitral annulus motion: Anatomical intimacy causing a good correlation? Exp. Clin. Cardiol. 10, 111–115 (2005). 19641670 PMC2716232

[80] M. C. Darnell, J. Y. Sun, M. Mehta, C. Johnson, P. R. Arany, Z. Suo, D. J. Mooney, Performance and biocompatibility of extremely tough alginate/polyacrylamide hydrogels. Biomaterials 34, 8042–8048 (2013). 23896005 10.1016/j.biomaterials.2013.06.061 PMC3775708

[81] J. Mark, *Physical Properties of Polymers Handbook* (Springer, 2007).

[82] B. Faurie, G. Souteyrand, P. Staat, M. Godin, C. Caussin, E. V. Belle, L. Mangin, P. Meyer, N. Dumonteil, M. Abdellaoui, J. Monségu, I. Durand-Zaleski, T. Lefèvre, Left ventricular rapid pacing via the valve delivery guidewire in transcatheter aortic valve replacement. J. Am. Coll. Cardiol. Intv. 12, 2449–2459 (2019). 10.1016/j.jcin.2019.09.029 31857014

[83] I. Daehnert, C. Rotzsch, M. Wiener, P. Schneider, Rapid right ventricular pacing is an alternative to adenosine in catheter interventional procedures for congenital heart disease. Heart 90, 1047–1050 (2004). 15310698 10.1136/hrt.2003.025650 PMC1768411

[84] R. Scarsini, R. A. Kotronias, G. L. D. Maria, S. Rajasundaram, T. J. Cahill, R. Brown, J. D. Newton, A. P. Banning, R. K. Kharbanda, Routine left ventricular pacing for patients undergoing transcatheter aortic valve replacement. Struct. Heart 3, 478–482 (2019).

[85] B. M. Jones, Y. Jobanputra, A. Krishnaswamy, S. Mick, M. Bhargava, B. L. Wilkoff, S. R. Kapadia, Rapid ventricular pacing during transcatheter valve procedures using an internal device and programmer: A demonstration of feasibility. Catheter. Cardiovasc. Interv. 95, 1042–1048 (2020). 31429191 10.1002/ccd.28450

[86] C. J. Chung, I. George, Emerging transcatheter therapies for tricuspid valve disease. JTCVS Open 2, 14–19 (2020). 36003678 10.1016/j.xjon.2020.04.003 PMC9390332

[87] D. I. Blusztein, R. T. Hahn, New therapeutic approach for tricuspid regurgitation: Transcatheter tricuspid valve replacement or repair. Front. Cardiovasc. Med. 10, 1080101 (2023). 36910541 10.3389/fcvm.2023.1080101 PMC9995444

[88] M. Mehr, M. Taramasso, C. Besler, T. Ruf, K. A. Connelly, M. Weber, E. Yzeiraj, D. Schiavi, A. Mangieri, L. Vaskelyte, H. Alessandrini, F. Deuschl, N. Brugger, H. Ahmad, L. Biasco, M. Orban, S. Deseive, D. Braun, K. P. Rommel, A. Pozzoli, C. Frerker, M. Näbauer, S. Massberg, G. Pedrazzini, G. H. Tang, S. Windecker, U. Schäfer, K. H. Kuck, H. Sievert, P. Denti, A. Latib, J. Schofer, G. Nickenig, N. Fam, S. von Bardeleben, P. Lurz, F. Maisano, J. Hausleiter, 1-year outcomes after edge-to-edge valve repair for symptomatic tricuspid regurgitation: Results from the trivalve registry. J. Am. Coll. Cardiol. Intv. 12, 1451–1461 (2019). 10.1016/j.jcin.2019.04.019 31395215

[89] S. Kodali, R. T. Hahn, M. F. Eleid, R. Kipperman, R. Smith, D. S. Lim, W. A. Gray, A. Narang, S. V. Pislaru, K. Koulogiannis, P. Grayburn, D. Fowler, K. Hawthorne, A. Dahou, S. H. Deo, P. Vandrangi, F. Deuschl, M. J. Mack, M. B. Leon, T. Feldman, C. J. Davidson; CLASP TR EFS Investigators, Feasibility study of the transcatheter valve repair system for severe tricuspid regurgitation. J. Am. Coll. Cardiol. 77, 345–356 (2021). 33509390 10.1016/j.jacc.2020.11.047

[90] F. Schlotter, M. Miura, K. P. Kresoja, B. Alushi, H. Alessandrini, A. Attinger-Toller, C. Besler, L. Biasco, D. Braun, E. Brochet, K. A. Connelly, S. de Bruijn, P. Denti, R. Estevez-Loureiro, N. Fam, M. Gavazzoni, D. Himbert, E. C. Ho, J. M. Juliard, D. Kalbacher, R. Kaple, F. Kreidel, A. Latib, E. Lubos, S. Ludwig, M. Mehr, V. Monivas, T. M. Nazif, G. Nickenig, G. Pedrazzini, A. Pozzoli, F. Praz, R. Puri, J. Rodés-Cabau, K. P. Rommel, U. Schäfer, J. Schofer, H. Sievert, G. H. L. Tang, H. Thiele, M. Unterhuber, A. Vahanian, R. S. von Bardeleben, M. von Roeder, J. G. Webb, M. Weber, M. G. Wild, S. Windecker, M. Zuber, J. Hausleiter, F. Maisano, M. B. Leon, R. T. Hahn, A. Lauten, M. Taramasso, P. Lurz, Outcomes of transcatheter tricuspid valve intervention by right ventricular function: A multicentre propensity-matched analysis. EuroIntervention 17, e343–e352 (2021). 33956637 10.4244/EIJ-D-21-00191 PMC9724849

[91] G. Russo, M. Taramasso, D. Pedicino, M. Gennari, M. Gavazzoni, A. Pozzoli, D. Muraru, L. P. Badano, M. Metra, F. Maisano, Challenges and future perspectives of transcatheter tricuspid valve interventions: Adopt old strategies or adapt to new opportunities? Eur. J. Heart Fail. 24, 442–454 (2022). 34894039 10.1002/ejhf.2398

[92] C. M. Yu, G. B. Bleeker, J. W. H. Fung, M. J. Schalij, Q. Zhang, E. E. V. D. Wall, Y. S. Chan, S. L. Kong, J. J. Bax, Left ventricular reverse remodeling but not clinical improvement predicts long-term survival after cardiac resynchronization therapy. Circulation 112, 1580–1586 (2005). 16144994 10.1161/CIRCULATIONAHA.105.538272

[93] F. S. O. Souza, D. M. Braile, R. W. Vieira, S. O. Rojas, N. L. Mortati, A. C. Rabelo, S. A. Oliveira, Technical aspects of lead implantation for left ventricle pacing through the coronary sinus, using anatomic radiology and intracavitary electrography in the cardiac resynchronization therapy. Braz. J. Cardiovasc. Surg. 20, 301–309 (2005).

[94] J. H. Gamble, N. Herring, M. Ginks, K. Rajappan, Y. Bashir, T. R. Betts, Procedural success of left ventricular lead placement for cardiac resynchronization therapy: A meta-analysis. JACC Clin. Electrophysiol. 2, 69–77 (2016). 29766856 10.1016/j.jacep.2015.08.009

[95] H. Mair, J. Sachweh, B. Meuris, G. Nollert, M. Schmoeckel, A. Schuetz, B. Reichart, S. Daebritz, Surgical epicardial left ventricular lead versus coronary sinus lead placement in biventricular pacing. Eur. J. Cardiothorac. Surg. 27, 235–242 (2005). 15691676 10.1016/j.ejcts.2004.09.029

[96] N. Bogale, K. Witte, S. Priori, J. Cleland, A. Auricchio, F. Gadler, A. Gitt, T. Limbourg, C. Linde, K. Dickstein, F. Fruhwald, B. Strohmer, M. Goethals, J. Vijgen, J. N. Trochu, D. Gras, M. Kindermann, C. Stellbrink, K. McDonnald, D. Keane, T. B. Gal, M. Glikson, M. Metra, M. Gasparini, A. Maass, L. Jordaens, M. Alings, A. I. Larsen, S. Færestrand, J. Delgado, L. Mont, H. Persson, H. P. B.-L. Rocca, S. Osswald, I. Squire, J. Morgan, The european cardiac resynchronization therapy survey: Comparison of outcomes between de novo cardiac resynchronization therapy implantations and upgrades. Eur. J. Heart Fail. 13, 974–983 (2011). 21771823 10.1093/eurjhf/hfr085

[97] A. S. Manolis, S. Koulouris, D. Tsiachris, Electrophysiology catheter-facilitated coronary sinus cannulation and implantation of cardiac resynchronization therapy systems. Hellenic J. Cardiol. 59, 26–33 (2018). 28778735 10.1016/j.hjc.2017.07.008

[98] D. Hofer, A. Breitenstein, Snare technique for coronary sinus cannulation in cardiac resynchronization therapy. Indian Pacing Electrophysiol. J. 20, 293–295 (2020). 33002591 10.1016/j.ipej.2020.09.004 PMC7691783

[99] J. L. D’Arcy, S. Coffey, M. A. Loudon, A. Kennedy, J. Pearson-Stuttard, J. Birks, E. Frangou, A. J. Farmer, D. Mant, J. Wilson, S. G. Myerson, B. D. Prendergast, Large-scale community echocardiographic screening reveals a major burden of undiagnosed valvular heart disease in older people: The oxvalve population cohort study. Eur. Heart J. 37, 3515–3522 (2016). 27354049 10.1093/eurheartj/ehw229 PMC5216199

[100] A. L. Axtell, V. Bhambhani, P. Moonsamy, E. W. Healy, M. H. Picard, T. M. Sundt, J. H. Wasfy, Surgery does not improve survival in patients with isolated severe tricuspid regurgitation. J. Am. Coll. Cardiol. 74, 715–725 (2019). 31071413 10.1016/j.jacc.2019.04.028 PMC8890054

[101] Y. Topilsky, J. M. Inojosa, G. Benfari, O. Vaturi, S. Maltais, H. Michelena, S. Mankad, M. Enriquez-Sarano, Clinical presentation and outcome of tricuspid regurgitation in patients with systolic dysfunction. Eur. Heart J. 39, 3584–3592 (2018). 30060125 10.1093/eurheartj/ehy434

[102] O. Stuge, J. Liddicoat, Emerging opportunities for cardiac surgeons within structural heart disease. J. Thoracic Cardiovasc. Surg. 132, 1258–1261 (2006). 10.1016/j.jtcvs.2006.08.049 17140937

[103] F. Alqahtani, C. O. Berzingi, S. Aljohani, M. Hijazi, A. Al-Hallak, M. Alkhouli, Contemporary trends in the use and outcomes of surgical treatment of tricuspid regurgitation. J. Am. Heart Assoc. 6, e007597 (2017). 29273638 10.1161/JAHA.117.007597 PMC5779056

[104] B. D. Prendergast, H. Baumgartner, V. Delgado, O. Gérard, M. Haude, A. Himmelmann, B. Iung, M. Leafstedt, J. Lennartz, F. Maisano, E. A. Marinelli, T. Modine, M. Mueller, S. R. Redwood, O. Rörick, C. Sahyoun, E. Saillant, L. Søndergaard, M. Thoenes, K. Thomitzek, M. Tschernich, A. Vahanian, O. Wendler, E. J. Zemke, J. J. Bax, Transcatheter heart valve interventions: where are we? where are we going? Eur. Heart J. 40, 422–440 (2019). 30608523 10.1093/eurheartj/ehy668

[105] N. V. Vasilyev, P. E. Dupont, P. J. D. Nido, Robotics and imaging in congenital heart surgery. Future Cardiol. 8, 285–296 (2012). 22413986 10.2217/fca.12.20 PMC3326442

[106] H. Rafii-Tari, C. J. Payne, G. Z. Yang, Current and emerging robot-assisted endovascular catheterization technologies: A review. Ann. Biomed. Eng. 42, 697–715 (2014). 24281653 10.1007/s10439-013-0946-8

[107] L. Asmarats, M. Taramasso, J. Rodés-Cabau, Tricuspid valve disease: Diagnosis, prognosis and management of a rapidly evolving field. Nat. Rev. Cardiol. 16, 538–554 (2019). 30988448 10.1038/s41569-019-0186-1

[108] M. Arnold, J. Haug, M. Landendinger, Tricuspid annuloplasty: Transcatheter approaches. Curr. Cardiol. Rep. 23, 139 (2021). 34410518 10.1007/s11886-021-01570-8 PMC8376705

[109] G. Muntané-Carol, A. Alperi, L. Faroux, E. Bédard, F. Philippon, J. Rodés-Cabau, Transcatheter tricuspid valve intervention: Coaptation devices. Front. Cardiovasc. Med. 7, 139 (2020). 32903754 10.3389/fcvm.2020.00139 PMC7438895

[110] M. Taramasso, R. T. Hahn, H. Alessandrini, A. Latib, A. Attinger-Toller, D. Braun, E. Brochet, K. A. Connelly, P. Denti, F. Deuschl, A. Englmaier, N. Fam, C. Frerker, J. Hausleiter, J. M. Juliard, R. Kaple, F. Kreidel, K. H. Kuck, S. Kuwata, M. Ancona, M. Malasa, T. Nazif, G. Nickenig, F. Nietlispach, A. Pozzoli, U. Schäfer, J. Schofer, R. Schueler, G. Tang, A. Vahanian, J. G. Webb, E. Yzeiraj, F. Maisano, M. B. Leon, The international multicenter trivalve registry: Which patients are undergoing transcatheter tricuspid repair? JACC: Cardiovascular Interventions 10, 1982–1990 (2017). 28982563 10.1016/j.jcin.2017.08.011

[111] J. Mesnier, A. Alperi, V. Panagides, E. Bédard, E. Salaun, F. Philippon, J. Rodés-Cabau, Transcatheter tricuspid valve interventions: Current devices and associated evidence. Prog. Cardiovasc. Dis. 69, 89–100 (2021). 34801577 10.1016/j.pcad.2021.11.007

[112] D. Kolte, S. Elmariah, Current state of transcatheter tricuspid valve repair. Cardiovasc. Diagn. Ther. 10, 89–97 (2020). 32175231 10.21037/cdt.2019.09.11 PMC7044094

[113] I. R. J. Webster, B. A. Jones, Design and kinematic modeling of constant curvature continuum robots: A review. Robot. Res. 29, 1661–1683 (2010).

[114] P. Rao, Q. Peyron, S. Lilge, J. Burgner-kahrs, How to model tendon-driven continuum robots and benchmark modelling performance. Front. Robot. AI 7, 630245 (2015). 10.3389/frobt.2020.630245 PMC7885639 33604355

[115] T. M. Vesely, T. K. Pilgram, Angioplasty balloon inflation pressures during treatment of hemodialysis graft-related stenoses. J. Vasc. Interv. Radiol. 17, 623–628 (2006). 16614144 10.1097/01.RVI.0000208988.28121.AB

