# Supplementary material for: A multifunctional soft robot for cardiac interventions
Source: Sci Adv. 2023 Oct 25;9(43):eadi5559. doi: 10.1126/sciadv.adi5559 (PMC10599628; doi:10.1126/sciadv.adi5559)
Supplement: Supplementary file 1 — Sections S1 to S13 Figs. S1 to S11 Table S1 Legends for movies S1 to S9 References [file sciadv.adi5559_sm.pdf]

Science Advances
AAAS

Supplementary Materials for

A multifunctional soft robot for cardiac interventions

Jacob Rogatinsky *et al.*

Corresponding author: Tommaso Ranzani, tranzani@bu.edu

Sci. Adv. **9**, eadi5559 (2023)
DOI: 10.1126/sciadv.adi5559

The PDF file includes:

Sections S1 to S13
Figs. S1 to S11
Table S1
Legends for movies S1 to S9
References

Other Supplementary Material for this manuscript includes the following:

Movies S1 to S9

S1 Clinical challenges in cardiac intervention

We have chosen to address two exemplar procedures in the RA because right side pathologies have, until recently, received less attention than left side pathologies. This trend is especially visible in the context of valve repair, wherein the TV has historically received the moniker of the "forgotten valve" (*61*). It was previously believed that TV pathologies were benign, leading clinicians to focus attention toward the mitral and aortic valves (*62*). However, recent evidence and advances in imaging modalities have shifted the climate, increasing the need for TV-targeted therapies, and greater attention to the right side in general. The selection of CS lead placement and TV annular anchor placement procedures is particularly significant because these procedures have fundamentally different requirements for success. CS lead placement requires a high degree of dexterity and navigability toward a small target, but a low degree of force generation. TV repair shifts the requirements toward effective force transmission while maintaining a need for high dexterity. Together, these two procedures require a combination of device characteristics that have not been investigated altogether: small size, a large but stable task space for operation inside the heart, and efficient force transmission.

Commercial devices have been developed to address these challenges in similar procedures. For example, systems like the Carto3 (Biosense Webster, Irvine, CA) (*3*) and the EnSite (Abbott, Chicago, IL) (*4*) are both used to create real-time intracardiac maps for RFA. When paired with conventional catheters, these systems provide interventionalists with a 3D workspace visualization without the need for harmful radiation from X-ray fluoroscopy. However, they fail to address the mobility issues faced by conventional catheters, and the generated cardiac maps do not account for motion. In clinical practice, clinicians regularly address intracardiac motion with respiration modulation and rapid ventricular cardiac pacing. Respiration modulation is a technique in which the patient's breathing frequency is changed for short periods of time, de-

creasing gross anatomical movements and allowing the clinician to increase their instrument's force consistency (*46*). Rapid ventricular pacing speeds up the heartbeat, turning the heart's normal low frequency high amplitude motion into a high frequency low amplitude vibration, and achieving similar results to respiration modulation (*82–85*). Alternatively, existing transcatheter devices for valve repair, including coaptation and annuloplasty devices, provide varying methods for proceduralists to apply forces on valvular tissue. Examples of coaptation devices include the Forma Repair System (Edwards Lifesciences, Irvine, CA), MitraClip System (Abbott Vascular, Santa Clara, CA), and Croivalve Duo (Croívalve, Dublin, Ireland) (*42,86,87*). Annuloplasty devices include the Trialign (Mitralign, Tewksbury, MA), Cardioband (Edwards Lifesciences, Irvine, CA), and Millipede IRIS (Boston Scientific, Marlborough, MA) (*42, 86*). These therapies face difficulties since their overall success relies on patient-specific device selection, especially given variability in underlying disease mechanisms. This approach leads to high dependence on the user's familiarity with the device, making procedures complex and time-consuming (*41, 88–91*).

Coronary Sinus Lead Placement Cardiac resynchronization therapy (CRT) has been shown to effectively treat heart failure, which is prevalent in up to 45% of adults over 45 years of age in the United States (*36*). CRT can be accomplished with multiple approaches, including epicardial lead placement, conduction system pacing, or CS lead placement. CS lead placement is typically recommended as the initial approach to establish CRT given the high likelihood of success and the plethora of data on CS lead efficacy and safety (*71, 92*). However, CS lead placement is not without its own challenges. Difficulties in lead advancement and CS cannulation have been observed in up to 25% of patients, with an overall failure rate of up to 5.4% (*93, 94*). Specific failure modes include CS dissection, loss of pacing capture, diaphragm stimulation, lead dislodgement, and incorrect lead positioning (*95*). The most common cause

of implant failure is navigating challenging anatomy in the RA like the Thebesian valve or a superior CS entrance, both of which vary between patients (*72, 73*). Additionally, a stable CS cannulation may expose patients to nearly thirty minutes of harmful X-ray fluoroscopy radiation during a procedure that can last several hours in total (*96, 97*).

Often, the catheters that allow interventionalists to access the heart in a minimally-invasive manner are also a source of difficulty when navigating the RA's complex anatomy. Generally, a proceduralist accesses the CS using a combination of guiding instruments, including guidewires, catheters, and sheaths, to incrementally advance toward the target site. However, conventional guide wires and catheters have low directable mobility, sometimes incorporating selective structural weakening to add up to one distal degree of freedom (DoF). Variations in anatomy (e.g., Thebesian valve, CS entrance) can make it difficult for these tools to produce consistent results across patients (*98*).

Tricuspid Valve Repair Valvular heart disease (VHD) exists in a large portion of the world population; for example, one study in the UK showed that 51% of their participants had some form of valve pathology (*99*). The global burden of VHD partially owes to the rising inequality in healthcare access, leading to some communities with aging populations, and other communities with inadequate access to medical care (*37*). Further, clinicians have historically focused on left-side VHD because right-side pathology, specifically TV regurgitation, was viewed as a secondary mechanism (*61*). It was previously assumed that functional regurgitation would improve after intervention on associated left-sided VHD, though data demonstrates ongoing residual regurgitation after treatment of mitral or aortic pathologies (*62*). Furthermore, description of TV regurgitation etiologies and the quantification of valve dysfunction were challenging issues given the high rates of postoperative morbidity and mortality (*100*). However, it is now known that TV regurgitation is a significant risk factor independent of left-side pathologies,

even when the severity is mild to moderate (*61, 74–76, 101*).

Despite the risks associated with untreated TR, open surgery presents a significant risk as well (*61, 102*). The in-hospital mortality rate is as high as 10.8%, in large part due to complications from the cardiopulmonary bypass (CPB) required to perform reconstructive repairs (*103*). Following the trend toward minimally-invasive procedures, transcatheter options are actively under investigation (*104–106*). Current transcatheter systems (Table S1) include annuloplasty devices, which shrink the diameter of the TV annulus, and coaptation devices, which use clips to approximate the TV leaflets (*43, 107–109*). For example, the Croívalve Duo uses a stent mechanism deployed in the SVC to stabilize a coaptation device in the tricuspid valve (*87*).

**Categories**	**Delivery Size**	**Utilization**	**Clinical Adoption**
**Valve Repair Platforms (*42, 86, 87*)**			
Millipede IRIS (Boston Scientific)	$8\ mm$	Annuloplasty	Human trials
Trialign (Mitralign)	$4.7\ mm$	Annuloplasty	Human trials
Cardioband (Edwards Lifesciences)	$8\ mm$	Annuloplasty	Human trials
TriCinch (4Tech)	$8\ mm$	Annuloplasty	Human trials
Forma (Edwards Lifesciences)	$8\ mm$	Coaptation	Human trials
Croívalve Duo (Croívalve)	N/A	Coaptation	Human trials
**Clinical Methods (*46, 82–85*)**			
Commercial Catheters	$1.3 - 8\ mm$	MIS	Yes
Rapid Ventricular Pacing	N/A	Motion reduction	Yes
Respiration Apnea	N/A	Motion reduction	Yes

Table S1: **Clinical devices and methods are characterized by size, utilization, and clinical adoption.** *Valve Repair Platforms*: Commercial valve repair devices for implantation in the tricuspid valve. *Clinical Methods*: Devices and methods used frequently in cardiac interventions.

Initial studies on different transcatheter approaches demonstrated their feasibility in treating severe TV regurgitation (*110, 111*). Despite the initial successes, beating-heart TV repair presents challenges due to the valve's motility (*77*). Additionally, the septal leaflet of the TV is next to the atrioventricular (AV) node of the conduction system, making device positioning crucial to avoid electrophysiological damage. They are further associated with residual levels of regurgitation, making them less effective than open surgery for severe cases of TR (*62*). Given

these challenges, transcatheter procedures remain less common than open surgery for valve repair, typically being performed on high-risk patients for whom open surgery would be too dangerous (*102*). There is a need for interventional TV repair approaches that avoid the complications associated with open-heart surgeries, enabling clinical translation and development of transcatheter procedures beyond patients contraindicated for surgery (*91, 112*).

S2 Soft manipulator modeling

We approximate the soft manipulator's imaginary central backbone as an arc of constant curvature and variable length. The following section discusses analytical and data-based relationships to find the manipulator's joint space parameters. Afterwards, we review the integration of these relationships with constant curvature (CC) kinematics to find the tip pose. The CC approximation is commonly used to model continuum robots by reducing infinite DoFs to a few parameters (*39, 113, 114*).

Analytical Relationships A single balloon is the smallest fundamental unit of this soft manipulator. The analytical relationship between the volume inside a single balloon and its height was first presented by the authors in a previous work (*69*). The goal is to find a relationship between the volume v input into the balloon, and the resultant height h of the balloon.

Geometric model: To derive a geometric model of a single balloon inflation, we start by looking at the balloon's vertical cross section. We fit circular, ellipsoid, and parabolic curves to the cross section, and determined by observation that the boundary of the inflated balloon is most accurately described by a parabola. Thus, three variables describe the balloon's boundary curve: volume v enclosed by the parabola as it's rotated around the y-axis, a coefficient α that dictates the width of the parabola, and the x-intercept ρ of the parabola as the balloon inflates. The following three equations can be solved for these three variables:

$$v = 2\pi \int_0^\rho x(h - \alpha x^2)dx \tag{S1}$$

$$\begin{aligned} y(x = \rho) = 0 = h - \alpha\rho^2 \\ h = \alpha\rho^2 \end{aligned} \tag{S2}$$

$$l = \int_0^\rho \sqrt{1 + (-2\alpha\rho)^2}dx \tag{S3}$$

Where l is the arc length of the curve, equivalent to the radius of the balloon when fully deflated. Solving this system of equations gives a relationship defining v with respect to h. After removing higher order terms and multiplying by two (since the original system only describes half a balloon), the resulting relationship is the following:

$$v = \frac{2\pi}{54l^2}\left(32h^5 - 24h^3l^2 + 18hl^4\right) \tag{S4}$$

This relationship cannot be analytically inverted to define h with respect to v. However, the inverse is necessary to feed the geometric model into a kinematic model. To work around this limitation, we generate a matrix of h and v values, where the h values are a linearly spaced vector, and the v values are generated from the model. Given sufficiently small increments between the h values, we can then use linear interpolation to match any volume used as input with its corresponding height.

The geometric model was compared with experimental data (Fig. S1A). We inflated a 3-DoF soft robot such that all the chambers had equal volumes at all times. The manipulator was inflated up to $0.5\ mL$ in ten increments, and this inflation was repeated 100 times for a total of 1000 data points. At each increment, an electromagnetic position tracking system (Northern Digital, Inc., Aurora) recorded the tip position relative to a sensor at the manipulator's base. The model and data demonstrate high concordance up to $\approx 0.15\ mL$, or $10\ mm$. After this point,

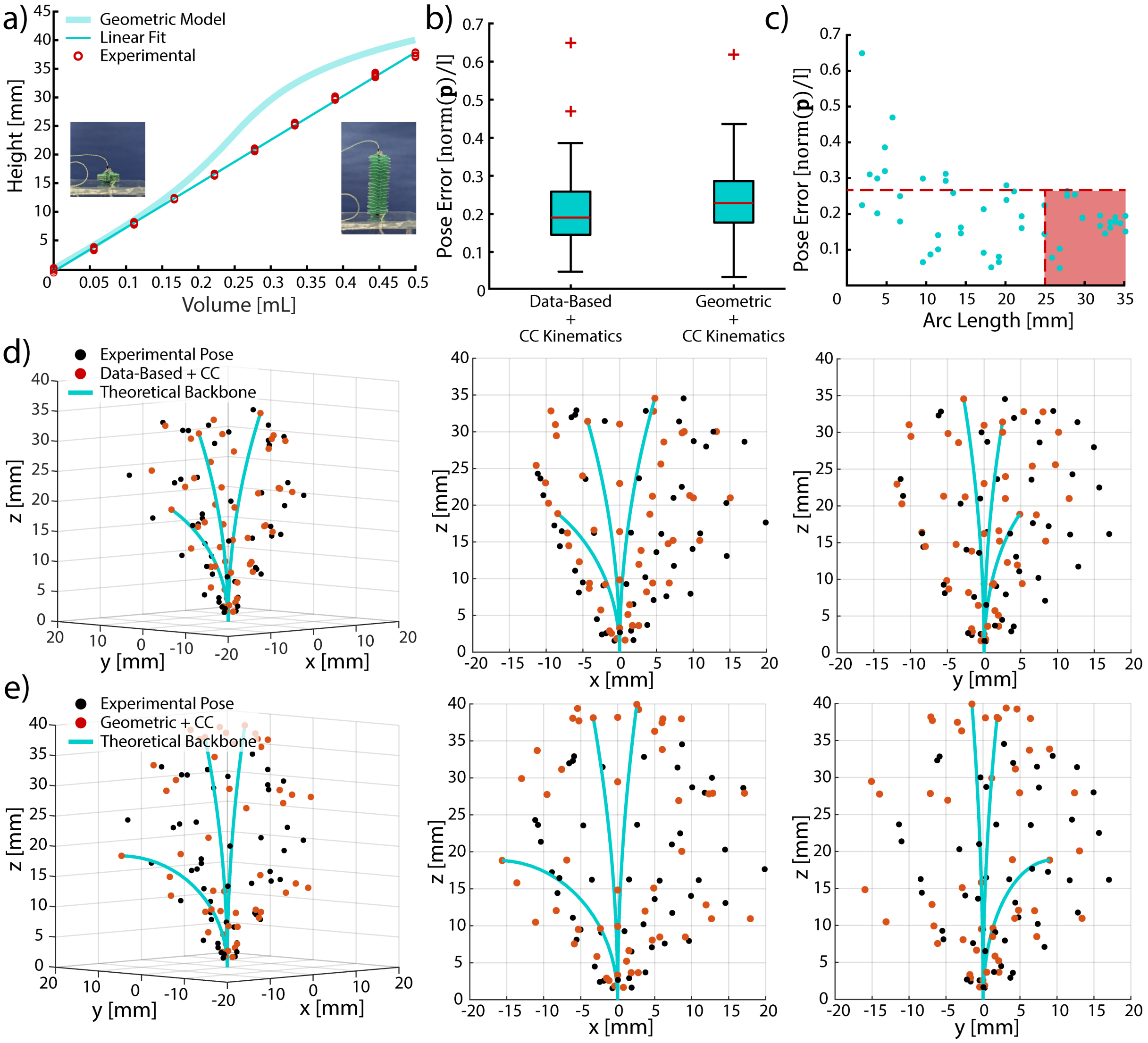

Figure S1: **Comparison of the relationship between soft manipulator input volume and resultant height with the experimental tip pose.** a) A soft manipulator was inflated vertically, and the resulting height values were compared to the geometric relationship. b) After integrating the results of the two volume-height relationships (i.e. geometric and data-based) with CC kinematics, the Euclidean distance between theoretical and experimental tip pose was normalized by corresponding robot arc length and presented with mean, IQR, range, and outliers. c) The error of the data-based relationship with CC kinematics is below 27% for large robot inflation values. d) The experimental data is compared with the data-based volume-height relationship and CC kinematics. e) The experimental data is compared with the geometric volume-height relationship and CC kinematics.

the geometric model over-predicts the soft manipulator's height. This overprediction may arise from several factors, including: 1) gravity hindering the inflation, 2) material strain hindering the inflation, and 3) manufacturing inconsistencies.

Data-driven model. Given the statistical significance of the data ($R^2 > 0.99$, p-value$= 0$) shown in Fig. S1A, we used the data to generate a linear model for the $v - h$ relationship as well. The accuracy of the geometric and data-driven models will be compared after integration with CC kinematics.

Constant Curvature Model Disregarding the effect of external forces, we exploit the CC assumption to generate a computationally efficient kinematic model for the soft robot. The CC assumption allows us to forego computationally expensive numerical methods by treating the imaginary central backbone as a semi-circular arc. CC kinematics also make it easy to model the variable arc length, since the arc length is directly described by the joint space parameters. Numerical methods, however, struggle to model excessive mesh deformations generated by a soft robot's large expansion ratio.

We begin to form the kinematic model with a robot-specific mapping from the joint space to the configuration space. For this soft robot, the joint space is a vector $\mathbf{q} = [l_1, l_2, l_3]^T$ of the individual chamber lengths, given by the analytical models from the previous section. The configuration space describes the robot's configuration with respect to the shape of the backbone arc, using the variables $\{\kappa, \phi, l\}$. κ is the backbone's curvature, ϕ is the out-of-plane rotation from the xz-plane, and l is the arc length. To find these arc parameters, we project the arc onto the xy-plane and find four equations to describe the geometry:

$$r_i = r - dcos\phi_i \tag{S5}$$

$$l_i = l - \theta d cos\phi_i \tag{S6}$$

$$\phi_i = 90° + 120°(i-1) - \phi \tag{S7}$$

$$\sum_{i=1}^{3} cos\phi_i = 0 \tag{S8}$$

By inspection, or by combining equations S6 and S8, we get:

$$l(\mathbf{q}) = \frac{l_1 + l_2 + l_3}{3} \tag{S9}$$

Evaluating equation S6 for the first two chambers and equating the results yields $\theta d = \frac{l_2 - l_1}{cos\phi_1 - cos\phi_2}$. Evaluating equation S6 for the second two chambers and equating the results yields $\theta d = \frac{l_3 - l_2}{cos\phi_2 - cos\phi_3}$. Equating these equations yields:

$$\phi(\mathbf{q}) = tan^{-1}\Big(\frac{\sqrt{3}(l_2 + l_3 - 2l_1)}{3(l_2 - l_3)}\Big) \tag{S10}$$

Finally, we determine by observation that $\theta = \kappa l = {}^{l_i}/_{r_i}$, so $r_i = {}^{l_i}/_{\kappa l}$. If we plug in equation S6, we get $\kappa = \frac{l - l_i}{l d cos\phi_1}$. Evaluating for a single chamber, $i = 1$, we get $\kappa = \frac{l_2 + l_3 - 2l_1}{(l_1 + l_2 + l_3) d sin\phi}$. Plugging in equation S10, we get:

$$\kappa(\mathbf{q}) = \frac{2\sqrt{l_1^2 + l_2^2 + l_3^2 - l_1 l_2 - l_1 l_3 - l_2 l_3}}{d(l_1 + l_2 + l_3)} \tag{S11}$$

Equations S9, S10, and S11 represent the robot's configuration space. We now perform a robot-independent mapping from the configuration space to the task space, which yields the robot's tip pose. The resulting transformation matrix is parameterized by the configuration space arc parameters. It is generated by multiplying an out-of-plane rotation by the angle ϕ with an in-plane transformation and rotation by the angle $\theta = \kappa l$:

$$T = \begin{bmatrix} R_z(\phi) & 0 \\ 0 & 1 \end{bmatrix} \begin{bmatrix} R_y(\theta) & \mathbf{p} \\ 0 & 1 \end{bmatrix} \quad \text{(S12)}$$

$R_z(\phi)$ and $R_y(\theta)$ are 3D rotation matrices and **p** is the tip position of the arc in the xz-plane, given by $\begin{bmatrix} r(1-cos\theta), 0, rsin\theta \end{bmatrix}^T$. Plugging these values in yields the transformation matrix:

$$T = \begin{bmatrix} cos\phi cos\theta & -sin\phi & cos\phi sin\theta & rcos\phi(1-cos\theta) \\ sin\phi cos\theta & cos\phi & sin\phi sin\theta & rsin\phi(1-cos\theta) \\ -sin\theta & 0 & cos\theta & rsin\theta \\ 0 & 0 & 0 & 1 \end{bmatrix} \quad \text{(S13)}$$

The issue with the transformation matrix in equation S13 is that the local coordinate frame at the tip always has its $\hat{e}_1$ basis always points toward the xy-plane. This is solved with a post-multiplication by the rotation matrix $R_z(-\phi)$ with zero translation, which fixes the orientation of the local tip frame relative to the base frame such that the local xy-plane does not rotate about the local z-plane. The resulting transformation matrix is as follows:

$$T_w = \begin{bmatrix} cos^2\phi(cos\theta-1)+1 & sin\phi cos\phi(cos\theta-1) & cos\phi sin\theta & rcos\phi(1-cos\theta) \\ sin\phi cos\phi(cos\theta-1) & cos^2\phi(1-cos\theta)+cos\theta & sin\phi sin\theta & rsin\phi(1-cos\theta) \\ -cos\phi sin\theta & -sin\phi sin\theta & cos\theta & rsin\theta \\ 0 & 0 & 0 & 1 \end{bmatrix} \quad \text{(S14)}$$

Model Validation The constant curvature assumption was integrated with both the geometric and data-based relationships between volume and height. The soft robot's experimental task space was recorded by sweeping through a pseudo-randomized subset of the full task space. The subset consisted of 50 volumetric input combinations ranging from $0.0 - 0.5\ mL$. The electromagnetic position tracking system recorded the tip position at each volumetric increment, generating an experimental point cloud representing the robot's tip pose at each input (Fig. S1D,E). Each point in the point cloud was correlated with the corresponding theoretical points and the error was determined by normalizing the Euclidean distance between the points by the arc

length of the robot at that configuration (Fig. S1B). The median error for the kinematics based on the data-based model is 19% and the median error for the kinematics based on the geometric model is 22%. Both have an IQR between 10% and 11%. However, the data-based model and kinematics are better at predicting tip pose at larger backbone arc lengths, with errors never rising above the IQR after exceeding $\approx 7\ mm$ in backbone arc length. The geometric model and kinematics did, however, present errors above the IQR throughout the whole range of backbone arc lengths. The data-based model's ability to predict tip pose at larger arc lengths makes it useful for the surgical application, where the points of interest (i.e., the CS and TV) can only be reached at large arc lengths. Given that the RA is statistically at least $35\ mm$ from base to apex (*44*), and the SVC entrance is on the opposite side from the CS ostium and TV annulus, the soft robot has to inflate at least $25\ mm$ to reach its targets. At that level of actuation, the median error is 17% with an IQR of 4% (Fig. S1C).

S3 Soft manipulator fabrication

The soft robotic manipulator is fabricated using a 2D additive process. Layers of thermoplastic elastomer (TPE) alternate with PTFE (Teflon), which serves as a mask to prevent TPE bonding in select areas. We designed and laser cut these layers such that three parallel stacks of balloons would be embedded into the laminate. After aligning and stacking the layers, they are subjected to heat and pressure ($140°C$, $70\ kPa$) for a total of forty minutes. The resulting laminate can radially fold to $1\ mm$ in thickness, and expand up to $4\ cm$. We also demonstrate a method to force a radial collapse of the manipulator, enabling deployment through peripheral vasculature. Herein, the soft robot is inflated and its ends are fixed. An applied vacuum then causes the manipulator to buckle and collapse in the radial direction. Using this process, the soft robot can morph from a nominal diameter of $15\ mm$ to a radially collapsed diameter of down to $4.5\ mm$.

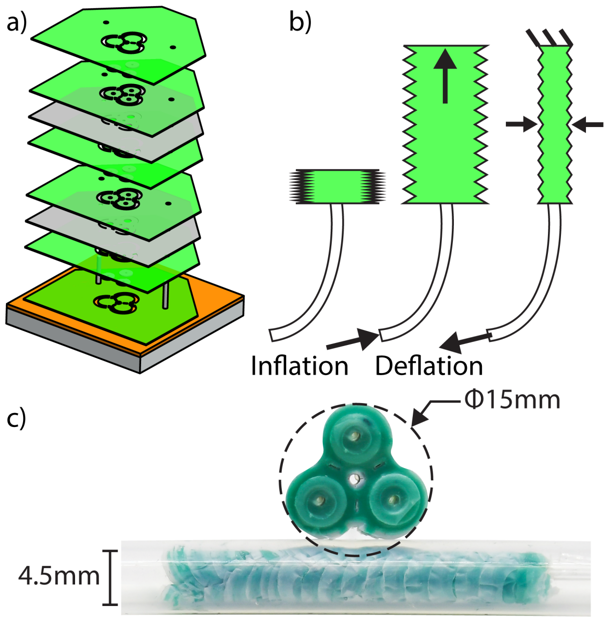

Figure S2: **Overview of soft robot fabrication and radial collapse.** a) The soft robot is manufactured with a 2D layered process using thermoplastic elastomer (TPE) and PTFE (Teflon). The TPE bonds to itself in the absence of the Teflon mask, thereby embedding an inflatable balloon geometry. b) The manipulator is radially collapsed by inflating all three chambers equally, fixing the tip so the length remains constant, and then pulling vacuum. c) As a result of the radial collapse, the manipulator's original diameter of $15\ mm$ shrinks to a minimum of $4.5\ mm$.

S4 Tool guide fabrication

The tool guide was fabricated using the same 2D additive method as the stabilization mechanism. Two layers of stainless steel sandwich a flexible layer of Kapton polyimide, and are coated on one side with a soft thermoplastic polyurethane (TPU). These layers are cut in a 5W UV laser, aligned and adhered with bioadhesive, and released from sacrificial material in the laser. The resulting laminate contains three distinct shaped lobes, enabling distinction under X-ray fluoroscopy. The lobes can also fold at their unidirectional flexure joints, thereby allowing the soft manipulator to radially buckle.

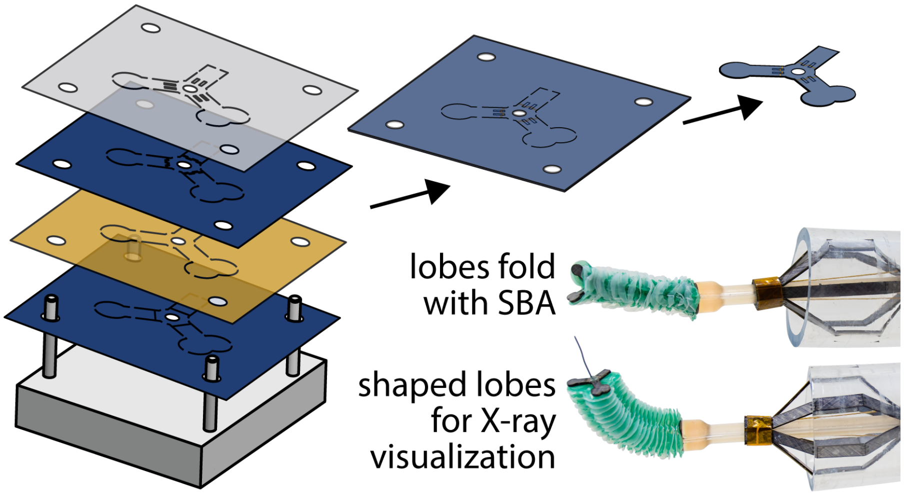

Figure S3: **Overview of tool guide fabrication and radial collapse.** The tool guide is manufactured with a 2D layered process using the same materials as the stabilization mechanism. Stainless steel provides rigidity, Kapton provides flexibility for the joints, and TPU provides softness for interaction with biological tissue. The resulting tool guide is adhered to the tip of the soft manipulator, and its three lobes can fold inward during the soft manipulator's radial collapse. The shaped, radiopaque lobes also allow a user to distinguish the different manipulator chambers under X-ray fluoroscopy.

S5 Stabilization mechanism *in-vitro* testing

Radial and axial force tests were performed on the stabilization mechanism in an *in-vitro* setting in addition to the *ex-vivo* test reported in the paper. In the *in-vitro* test, the stabilization was deployed in a lumen made of TPU, rather than an explanted porcine SVC. The device withstood $1.67 \pm 0.19\ N$ of force for a radial displacement of $2\ mm$, and $2.19 \pm 0.27\ N$ of force for an axial displacement of $0.1\ mm$.

The stabilization withstood more radial force with less displacement in an *in-vitro* setting, likely due to the inextensibility of the TPU relative to the SVC. Conversely, it withstood more axial force in the *ex-vivo* case because the linkages could slightly distend the SVC and shape-lock, while the TPU lumen did not allow any material distension. This caused the stabilization mechanism to slip after a considerably shorter displacement, and at a lower force.

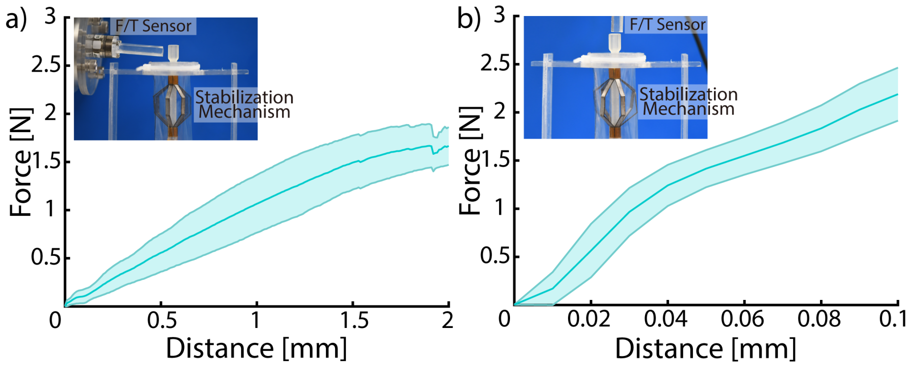

Figure S4: **Overview of radial (a) and axial (b) force tests on the stabilization mechanism.** The stabilization mechanism was deployed in a TPU lumen and its tip was displaced while recording the corresponding force. a) The stabilization mechanism was radially indented $n = 5$ times. One trial was discarded due to device slip. For a radial displacement of $2\ mm$, the stabilization withstood $1.67 \pm 0.19\ N$ of force. b) The stabilization mechanism was axially indented $n = 5$ times. For an axial displacement of $0.1\ mm$, the stabilization withstood $2.19 \pm 0.27\ N$ of force.

S6 SVC microscope analysis

Twelve individual microscope slides were stitched together using the panorama function in Adobe Photoshop (Fig. S5). The images were taken using a Nikon lens adapter for a Zeiss benchtop lab microscope, at $1/100^{th}\ s$ shutter speed and 1200 ISO. The resulting panoramic image had its background removed and replaced with a solid black background. An experienced clinician analyzed the image for signs of damage from the stabilization mechanism's extended deployment during a $4\ h$ *ex-vivo* test. Aside from an aberration made by forceps, likely during pre-test sample preparation, it was concluded that the stabilization mechanism did not cause any damage.

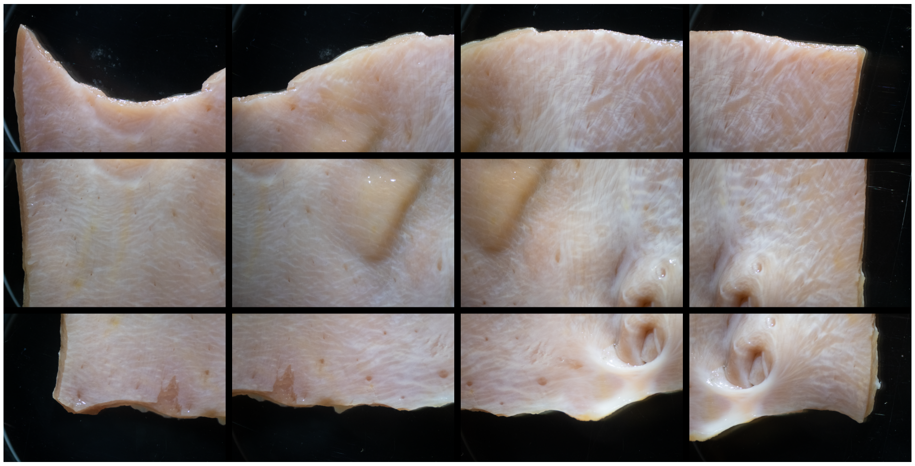

Figure S5: **Microscope slides were stitched together in Adobe Photoshop to generate a detailed image of the dissected SVC.** The resulting image was analyzed by an experienced clinician to determine whether the stabilization mechanism's deployment caused damage. Original microscope slides are shown in the figure.

S7 Soft manipulator controller

We implemented an open loop controller for the soft manipulator, in which user input from a Wii Classic remote controller directly modulates the volumes of the actuator chambers (Fig. S6A,B). This method was chosen because clinicians are already accustomed to guiding catheters with visual feedback from X-ray fluoroscopy. Therefore, direct control over the robot's DoFs maintains consistency with the surgical workflow. The user can directly control each chamber by visual inspection since each colored button on the Wii remote corresponds with a lobe of the tool guide (Fig. S6B,C). The patterned lobes are also discernible under X-ray fluoroscopy (Fig. S6C), allowing the user to navigate the soft manipulator without a direct line of sight. Further, X-ray fluoroscopy allows the user to visualize the location of a radiopaque tool, such as a guidewire or catheter, within the device Fig.S6C

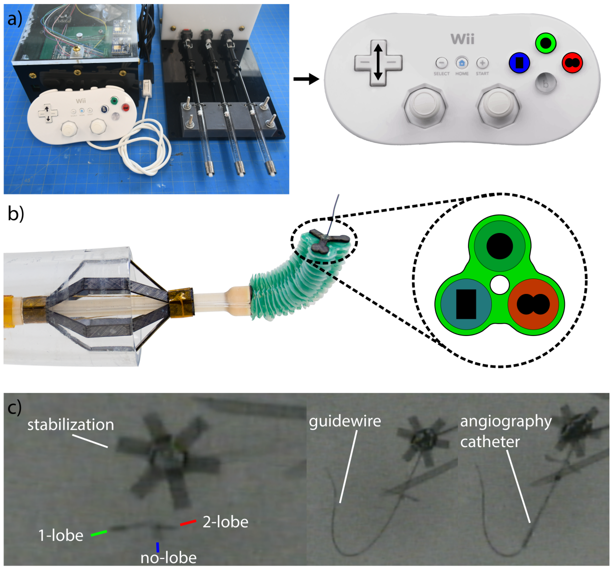

Figure S6: **Control hardware overview.** a) A Wii Classic controller receives user input. b) Each colored button on the controller corresponds with a chamber of the soft manipulator. c) Under X-ray fluoroscopy, the actuator chambers of the soft manipulator are discernible via the tool guide's patterned lobes. The insertion of conventional tools (i.e. angiography catheter and guidewire) through the robot's central working channel further enhances visualization.

S8 Tricuspid valve puncture feasibility and safety

We performed a puncture test on the TV annular tissue to determine the force required for puncture. We dissected an *ex-vivo* porcine tricuspid valve and laid the sample on a sheet of acrylic. We then connected a $0.25\ mm$ needle to a force sensor (ATI Industrial Automation Inc., NANO17) and linearly punctured the annular tissue at five separate locations while recording the maximum force. The punctures were performed by a six-axis robotic arm (Universal Robots) to ensure a consistent force vector. We found that $0.84 \pm 0.12\ N$ were sufficient to puncture the annular tissue, which is consistent with the $\approx 1\ N$ force required to puncture septal tissue (*47*).

In a clinical setting, puncturing the TV annulus would transmit a significant amount of stress to the SVC via the stabilization mechanism. To determine whether this force generation would

impart a harmful level of stress on the SVC, we analyzed the endothelium after a 4 *hr ex-vivo* test. During this test, we performed successful CS cannulations and punctured the TV annulus. The stabilization mechanism was repeatedly deployed for the duration of the procedure. Despite the prolonged stress from stabilization and force generation from the annular puncture trials, no damage was found after inspection by a clinician (Fig.S5).

S9 Interventional device dimensions

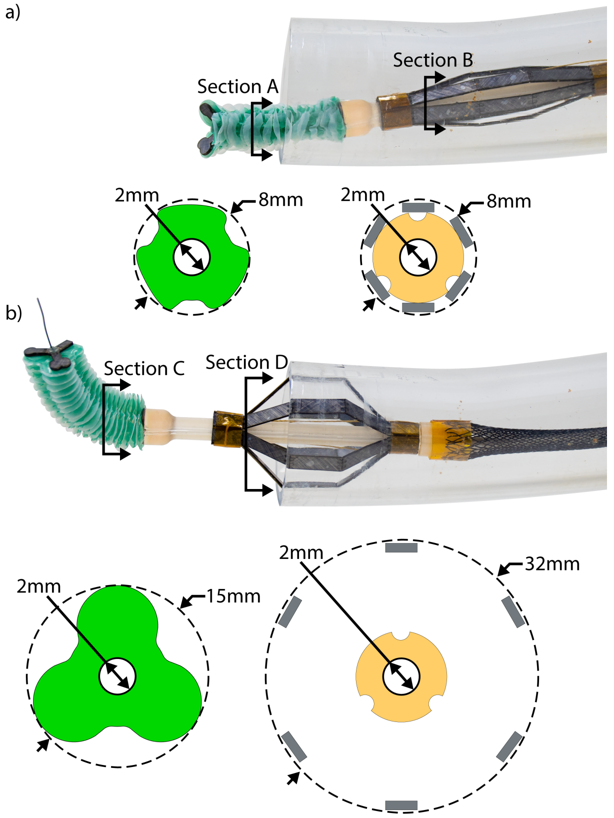

Figure S7: **Device dimensions in collapsed and expanded states.** a) *Collapsed*: Sections A and B show the 2 mm or 6 Fr central lumen, and the 8 mm or 24 Fr collapsed components. b) *Actuated*: Sections C and D show the 6 Fr central lumen, the 15 mm expanded manipulator, and the 32 mm expanded stabilization mechanism.

A 6 Fr length of tubing runs through the entire device, giving the operator a direct working

channel from the point of entry to the tip of the soft manipulator. Regardless of the collapse state of the manipulator and stabilization mechanism, the central lumen retains its 6 Fr diameter (Fig. S7). When collapsed for insertion, as shown in Sections A and B, the outer diameter of all device components can collapse down to $8\ mm$. This allows them to fit in a 24 Fr introducer sheath. When expanded for deployment, the manipulator reaches a nominal outer diameter of $15\ mm$ (Section C), and the stabilization mechanism reaches a maximum outer diameter of $32\ mm$ (Section D). These expanded diameters allow the device to exploit the larger intracardiac workspace after delivery through the narrower vascular workspace.

S10 Soft manipulator burst

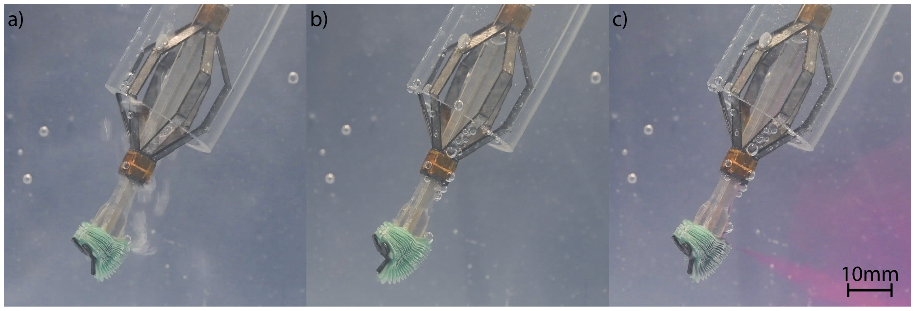

Figure S8: **Soft manipulator during active leaking.** a) When actuated with air, a considerable embolism is generated. b) When actuated with saline, minimal embolism is generated. c) When actuated with dyed water, fluid flow can be visualized.

The soft manipulator is actuated with saline solution to minimize risks associated with a fluid leak inside the bloodstream. If air were used as the working fluid, a leak would lead to a significant embolism, putting the patient at a high risk for complications. Using saline, the risk of an embolism is minimized. Additionally, interventionalists flush guidewires, angiography catheters, and introducer sheaths with up to $10\ mL$ of saline at a time for better lubrication. Since each actuating chamber of the soft manipulator is connected to a closed system with only $1\ mL$ of saline, a potential leak would release considerably less saline into the bloodstream than

regular tool flushing.

The risk of a leak occurring during a procedure is also low. In addition to each manipulator being tested before use, the maximum working pressure of the actuating chambers was found to be $78.9 \pm 5.1\ kPa$. This is less than the $367 \pm 18\ kPa$ burst pressure measured in a previous work (*69*), and considerably less than the $1700\ kPa$ working pressure of commercial balloon catheters (*115*). The maximum working pressure is programmatically enforced by limiting the actuation of the linear motors driving each fluidic syringe.

S11 Interventional device insertion and removal

The device is inserted into the right atrium via a 24 Fr introducer sheath. To fit the device through the sheath, the soft manipulator must be radially collapsed, as shown in Figure S9A. The manipulator is inflated and held in an extended state with a stiff wire through its central working channel, and vacuum applied to each actuating chamber causes them to buckle radially. The radial collapse allows the device to fit in the 24 Fr, or $8\ mm$, introducer sheath (Fig. S9B). Once inside the right atrium, the user can deploy the stabilization mechanism (Fig. S9C-i), infuse the manipulator with fluid (Fig. S9C-ii), and remove the fluid to regain axial folding (Fig. S9C-iii). The soft manipulator can then be actuated to reach any point in the right atrium (Fig. S9C-iv). To remove the device, the manipulator is radially collapsed using the same method previously described (Fig. S9C-v), and then retracted back through the introducer sheath. Because the introducer sheath has a fixed diameter, the soft manipulator further collapsed radially to conform with the sheath. As a result, the removed device remains intact in the radially collapsed state (Fig. S9C-vi).

After two trials of device insertion, deployment, and removal, the SVC was dissected and analyzed under a microscope for signs of trauma. The image in Figure S9D was merged together in Adobe Lightroom using the same method described in Section S6. With clinical collabora-

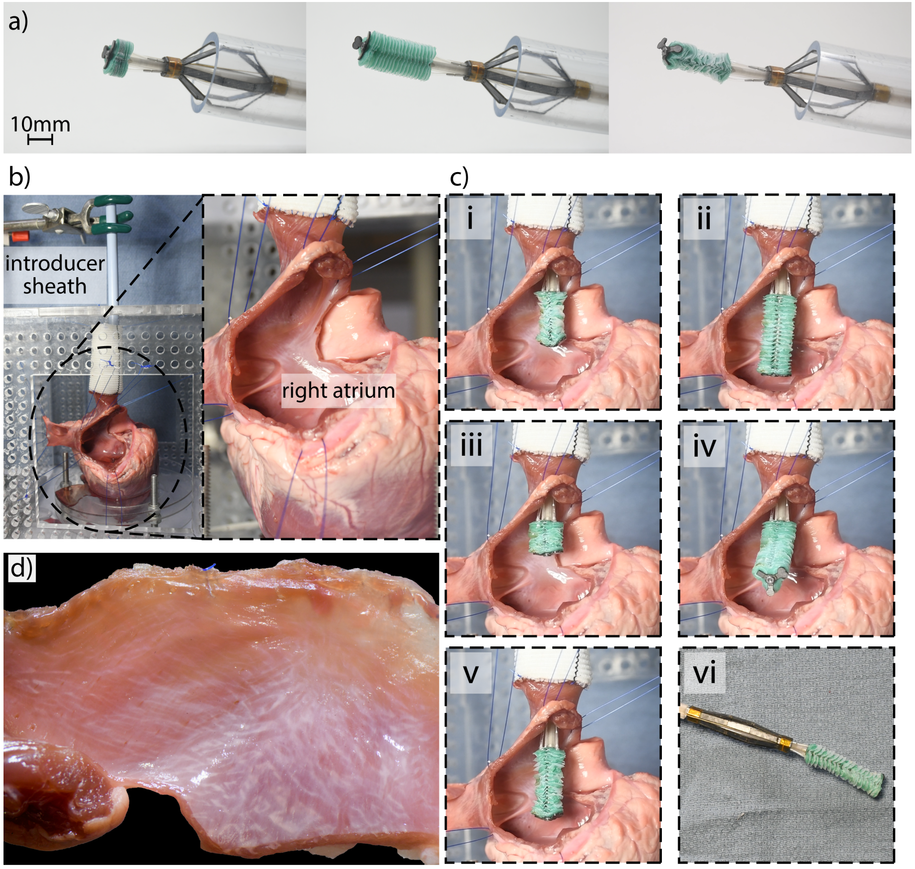

Figure S9: **Device insertion, deployment, actuation, and removal in *ex-vivo* porcine heart.** a) The device is radially collapsed in preparation for insertion. b) The *ex-vivo* porcine heart with an introducer sheath leading to the proximal SVC and RA. c) A series of steps shows the device (i) inserted, (ii-iv) deployed and actuated, (v) radially collapsed, and (vi) removed. d) The dissected SVC was undamaged after device operation.
Abbreviations: SVC – superior vena cava; RA – right atrium

tors from Boston Children's Hospital (Boston, MA, USA), it was determined that the device deployment did not cause trauma to the endothelium.

S12 Interventional device pressure drop

We repeated the flow pressure test from Figure 2D, but with the full interventional device inside a larger test section meant to simulate the right atrium. In both of these tests, we generate a

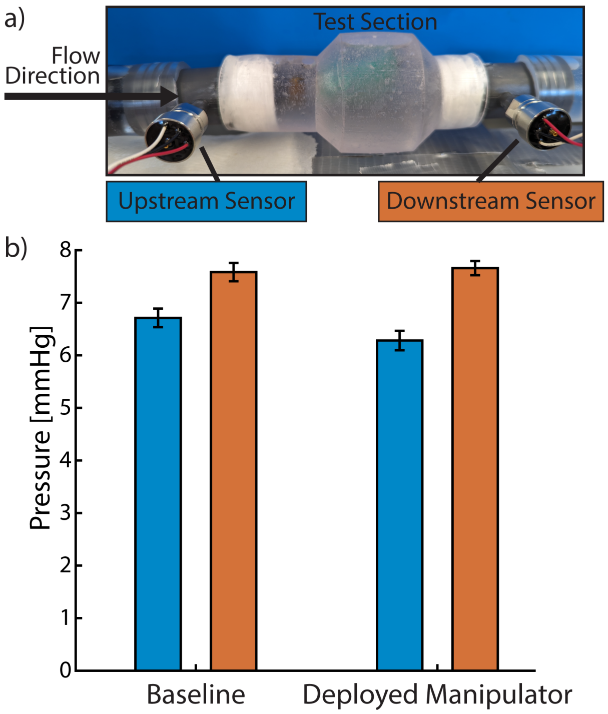

Figure S10: **Interventional robot fluidic resistance measurements.** a) The device is deployed in a $50\ mm$ diameter tube to simulate the size of the right atrium. b) The downstream pressures in the baseline and test trials are $7.58\ mmHg$ and $7.66\ mmHg$, respectively.

constant upstream pressure. Therefore, the downstream pressure should only fluctuate if the fluidic resistance caused by deploying the interventional device is large enough to reduce the downstream flowrate. If the fluidic resistance is negligible, we should see a negligible change in downstream pressure.

Indeed, the downstream pressure with the deployed manipulator of $7.66\ mmHg$ is only 1.1% different from the baseline downstream pressure. This indicates that deploying the device and inflating the soft manipulator in the right atrium generates negligible resistance in the flow.

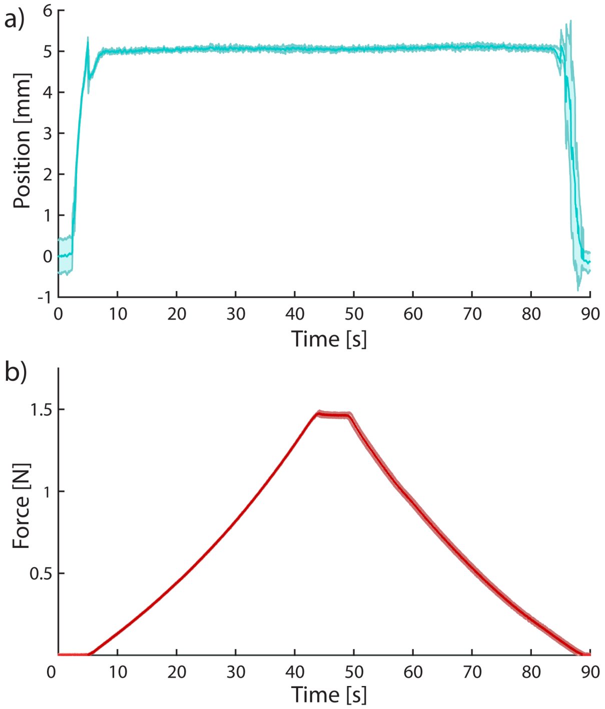

Figure S11: **Soft manipulator force characterization.** a) The isolated soft manipulator was inflated $5\ mm$ to press on a force sensor. b) The soft manipulator generates a maximum force on the force sensor for $5\ s$ before deflating.

S13 Viscoelastic effects under loading

We performed a time-dependent force characterization on the isolated soft manipulator. At $5\ mm$ of inflation, the manipulator began to press on a force sensor until it reached a maximum force generation of $1.47\ N$. Over $5\ s$, the force decreased by just 1.1%, with half of that decrease occurring in the first $1.2\ s$. This force decrease is indicative of negligible viscoelastic behavior in the manipulator's constituent materials at the loading conditions faced in a clinical environment. We can therefore conclude that the majority of the force decrease seen in Figure 5 and Figure 8D arises from strain creep in the superior vena cava (*67*).

S14 Movie M1

The integrated robotic platform performs a tool guidance task to demonstrate its dexterity. An acrylic sheet with five holes, each with a diameter of $10\ mm$, was placed $4\ cm$ from the base of the soft manipulator once it was deployed. The manipulator then guides a guidewire through all the holes while avoiding contact with them.

The platform also performs a task to demonstrate its ability to generate forces. After deployment, the soft manipulator presses against a syringe, which is connected to another soft robotic manipulator. Successful indentation of the syringe's plunger causes the downstream soft robot to inflate.

S15 Movie M2

The integrated robotic platform is deployed in an *ex-vivo* porcine heart sample, demonstrating the soft manipulator's radial collapse upon deployment. The user then directs the robot toward the CS to successfully cannulate it with a guidewire.

S16 Movie M3

Representative results from the CS cannulation test are shown. These results are also compared with the control trial, wherein an experienced clinician performed the same task using a conventional catheter and guidewire. Successful cannulation is achieved with both the conventional catheter and the robot. The video shows how the robot allows controlling the placement of the catheter in a more intentional way with respect to using a conventional catheter.

S17 Movie M4

Representative results from the TV annular puncture task are shown for a static *in-vitro* setup. The video demonstrates the robot braced on a TPU tube puncturing a silicone ring that represents the tricuspid annulus.

S18 Movie M5

Representative results from the TV annular puncture task are shown for an *ex-vivo* setup. This test is performed for both a static TV sample and a motile TV sample. This video demonstrates the soft robot's material damping as it maintains constant contact with a motile target.

S19 Movie M6

Results from the contact force demonstration are shown. The robot is deployed in an explanted porcine SVC and directed toward a moving acrylic target. The robot maintains contact and the resulting contact forces are displayed, demonstrating the effect of passive material damping.

S20 Movie M7

The video shows the interventional robot cannulating the CS with a guidewire and angiography catheter under fluoroscopic guidance. The robot was deployed in an *ex-vivo* porcine heart submersed in water, and the operators could successfully reach the CS target using external imaging guidance.

S21 Movie M8

The video shows testing of the robot under pulsatile flow conditions simulating the flow parameters in the right atrium using the ViVitro Labs SuperPump. We generated flow in $\approx 40\ mm$

diameter tubing, with systolic and diastolic pressures of $8\ mmHg$ and $4\ mmHg$, respectively. With a 60 bpm frequency and $30\ mL$ of water delivered each cycle, we calculated a maximum flow rate of $30mL/s$. Throughout the ten minutes of the video, the soft manipulator did not move with respect to the flow.

S22 Movie M9

The video shows a leak in the soft manipulator when actuated with different fluids. Air generates embolism, while water does not.

REFERENCES AND NOTES

1. P. E. Dupont, B. J. Nelson, M. Goldfarb, B. Hannaford, A. Menciassi, M. K. O'Malley, N. Simaan, P. Valdastri, G. Z. Yang, A decade retrospective of medical robotics research from 2010 to 2020. *Sci. Robot.* **6**, eabi8017 (2021).

2. C. Freschi, V. Ferrari, F. Melfi, M. Ferrari, F. Mosca, A. Cuschieri, Technical review of the da vinci surgical telemanipulator. *Int. J. Med. Robot.* **9**, 396–406 (2013).

3. L. Nagaraju, D. Menon, P. F. Aziz, Use of 3d electroanatomical navigation (carto-3) to minimize or eliminate fluoroscopy use in the ablation of pediatric supraventricular tachyarrhythmias. *Pacing Clin. Electrophysiol.* **39**, 574–580 (2016).

4. M. Giaccardi, G. Mascia, A. P. Perini, A. Giomi, S. Cartei, M. Milli, Long-term outcomes after zero x-ray arrhythmia ablation. *J. Interv. Card. Electrophysiol.* **54**, 43–48 (2019).

5. R. Tarwala, L. D. Dorr, Robotic assisted total hip arthroplasty using the MAKO platform. *Curr. Rev. Musculoskelet. Med.* **4**, 151–156 (2011).

6. G. Fagogenis, M. Mencattelli, Z. Machaidze, B. Rosa, K. Price, F. Wu, V. Weixler, M. Saeed, J. E. Mayer, P. E. Dupont, Autonomous robotic intracardiac catheter navigation using haptic vision. *Sci. Robot.* **4**, eaaw1977 (2019).

7. A. Ataollahi, I. Berra, N. V. Vasilyev, Z. Machaidze, P. E. Dupont, Cardioscopic tool-delivery instrument for beating-heart surgery. *IEEE/ASME Trans. Mechatron.* **21**, 584–590 (2016).

8. N. V. Vasilyev, A. H. Gosline, A. Veeramani, M. T. Wu, G. P. Schmitz, R. T. Chen, V. Arabagi, P. J. D. Nido, P. E. Dupont, Tissue removal inside the beating heart using a robotically delivered metal mems tool. *Int. J. Robot. Res.* **34**, 236–247 (2015).

9. X. Zhang, M. C. Palumbo, F. Perico, M. Magro, A. Fortuna, T. Magni, E. Votta, A. Segato, E. D. Momi, Robotic actuation and control of a catheter for structural intervention cardiology, in *IEEE International Conference on Intelligent Robots and Systems (IROS)* (IEEE, 2022), pp. 5907–5913 (2022).

10. P. M. Loschak, A. Degirmenci, C. M. Tschabrunn, E. Anter, R. D. Howe, Automatically steering cardiac catheters in vivo with respiratory motion compensation. *Int. J. Robot. Res.* **39**, 586–597 (2020).

11. R. Qi, N. U. Nayar, J. P. Desai, Telerobotic transcatheter delivery system for mitral valve implant. *IEEE Robot. Autom. Lett.* 1–8 (2023).

12. M. Rox, D. S. Esser, M. E. Smith, T. E. Ertop, M. Emerson, F. Maldonado, E. A. Gillaspie, A. Kuntz, R. J. Webster, Toward continuum robot tentacles for lung interventions: Exploring folding support disks. *IEEE Robot. Autom. Lett.* **8**, 3494–3501 (2023).
13. Y. Kim, S. S. Cheng, M. Diakite, R. P. Gullapalli, J. M. Simard, J. P. Desai, Toward the development of a flexible mesoscale mri-compatible neurosurgical continuum robot. *IEEE Transact. Robot.* **33**, 1386–1397 (2017).
14. J. W. Martin, L. Barducci, B. Scaglioni, J. C. Norton, C. Winters, V. Subramanian, A. Arezzo, K. L. Obstein, P. Valdastri, Robotic autonomy for magnetic endoscope biopsy. *IEEE Trans. Med. Robot. Bionics* **4**, 599–607 (2022).
15. M. Cianchetti, T. Ranzani, G. Gerboni, T. Nanayakkara, K. Althoefer, P. Dasgupta, A. Menciassi, Soft robotics technologies to address shortcomings in today's minimally invasive surgery: The stiff-flop approach. *Soft Robotics* **1**, 122–131 (2014).
16. M. Cianchetti, C. Laschi, A. Menciassi, P. Dario, Biomedical applications of soft robotics. *Nat. Rev. Mater.* **3**, 143–153 (2018).
17. M. Runciman, A. Darzi, G. P. Mylonas, Soft robotics in minimally invasive surgery. *Soft Robot.* **6**, 423–443 (2019).
18. K. Ikuta, Y. Matsuda, D. Yajima, Y. Ota, Pressure pulse drive: A control method for the precise bending of hydraulic active catheters. *IEEE/ASME Transact. Mechatron.* **17**, 876–883 (2012).
19. M. Li, R. Obregon, J. J. Heit, A. Norbash, E. W. Hawkes, T. K. Morimoto, Vine catheter for endovascular surgery. *IEEE Trans. Med. Robot. Bionics* **3**, 384–391 (2021).
20. C. Fischer, Q. Boehler, B. J. Nelson, Using magnetic fields to navigate and simultaneously localize catheters in endoluminal environments. *IEEE Robot. Autom. Lett.* **7**, 7217–7223 (2022).
21. Y. Kim, G. A. Parada, S. Liu, X. Zhao, Ferromagnetic soft continuum robots. *Sci. Robot.* **4**, eaax7329 (2019).
22. T. L. Thomas, J. Sikorski, G. K. Ananthasuresh, V. K. Venkiteswaran, S. Misra, Design, sensing, and control of a magnetic compliant continuum manipulator. *IEEE Trans. Med. Robot. Bionics* **4**, 910–921 (2022).
23. E. T. Roche, M. A. Horvath, I. Wamala, A. Alazmani, S. E. Song, W. Whyte, Z. Machaidze, C. J. Payne, J. C. Weaver, G. Fishbein, J. Kuebler, N. V. Vasilyev, D. J. Mooney, F. A. Pigula, C. J. Walsh, Soft robotic sleeve supports heart function. *Sci. Transl. Med.* **9**, eaaf3925 (2017).

24. C. J. Payne, I. Wamala, D. Bautista-Salinas, M. Saeed, D. V. Story, T. Thalhofer, M. A. Horvath, C. Abah, P. J. D. Nido, C. J. Walsh, N. V. Vasilyev, Soft robotic ventricular assist device with septal bracing for therapy of heart failure. *Sci. Robot.* **2**, eaan6736 (2017).
25. N. A. Patronik, C. N. Riviere, S. E. Qarra, M. A. Zenati, The heartlander: A novel epicardial crawling robot for myocardial injections. *Int. Congr. Ser.* **1281**, 735–739 (2005).
26. N. A. Patronik, M. A. Zenati, C. N. Riviere, C. N. Riviere, Preliminary evaluation of a mobile robotic device for navigation and intervention on the beating heart. *Comput. Aided Surg.* **10**, 225–232 (2010).
27. Y. Kim, E. Genevriere, P. Harker, J. Choe, M. Balicki, R. W. Regenhardt, J. E. Vranic, A. A. Dmytriw, A. B. Patel, X. Zhao, Telerobotic neurovascular interventions with magnetic manipulation. *Sci. Robot.* **7**, eabg9907 (2022).
28. T. Amadeo, D. V. Lewen, T. Janke, T. Ranzani, A. Devaiah, U. Upadhyay, S. Russo, Soft robotic deployable origami actuators for neurosurgical brain retraction. *Front. Robot. AI* **8**, 731010 (2022).
29. D. Van Lewen, T. Janke, H. Lee, R. Austin, E. Billatos, S. Russo, A millimeter-scale soft robot for tissue biopsy procedures. *Adv. Intell. Syst.* **5**, 2200326 (2023).
30. M. McCandless, A. Perry, N. DiFilippo, A. Carroll, E. Billatos, S. Russo, A soft robot for peripheral lung cancer diagnosis and therapy. *Soft Robot.* **9**, 754–766 (2021).
31. G. Pittiglio, P. Lloyd, T. D. Veiga, O. Onaizah, C. Pompili, J. H. Chandler, P. Valdastri, Patient-specific magnetic catheters for atraumatic autonomous endoscopy. *Soft Robot.* **9**, 1120–1133 (2022).
32. M. McCandless, A. Gerald, A. Carroll, H. Aihara, S. Russo, A soft robotic sleeve for safer colonoscopy procedures. *IEEE Robot. Autom. Lett.* **6**, 5292–5299 (2021).
33. D. Son, H. Gilbert, M. Sitti, Magnetically actuated soft capsule endoscope for fine-needle biopsy. *Soft Robot.* **7**, 10–21 (2020).
34. S. Russo, T. Ranzani, C. J. Walsh, R. J. Wood, An additive millimeter-scale fabrication method for soft biocompatible actuators and sensors. *Adv. Mater. Technol.* **2**, 1700135 (2017).
35. T. Ranzani, G. Gerboni, M. Cianchetti, A. Menciassi, A bioinspired soft manipulator for minimally invasive surgery. *Bioinspir. Biomim.* **10**, 035008 (2015).
36. C. W. Tsao, A. W. Aday, Z. I. Almarzooq, A. Alonso, A. Z. Beaton, M. S. Bittencourt, A. K. Boehme, A. E. Buxton, A. P. Carson, Y. Commodore-Mensah, M. S. Elkind, K. R. Evenson, C. Eze-Nliam, J. F. Ferguson, G. Generoso, J. E. Ho, R. Kalani, S. S. Khan, B. M. Kissela, K. L.

Knutson, D. A. Levine, T. T. Lewis, J. Liu, M. S. Loop, J. Ma, M. E. Mussolino, S. D. Navaneethan, A. M. Perak, R. Poudel, M. Rezk-Hanna, G. A. Roth, E. B. Schroeder, S. H. Shah, E. L. Thacker, L. B. Vanwagner, S. S. Virani, J. H. Voecks, N. Y. Wang, K. Yaffe, S. S. Martin, Heart disease and stroke statistics–2022 update: A report from the american heart association. *Circulation* **145**, E153–E639 (2022).

37. S. Coffey, R. Roberts-Thomson, A. Brown, J. Carapetis, M. Chen, M. Enriquez-Sarano, L. Zühlke, B. D. Prendergast, Global epidemiology of valvular heart disease. *Nat. Rev. Cardiol.* **18**, 853–864 (2021).
38. Y. Wang, R. Bellomo, Cardiac surgery-associated acute kidney injury: Risk factors, pathophysiology and treatment. *Nat. Rev. Nephrol.* **13**, 697–711 (2017).
39. J. Burgner-Kahrs, D. C. Rucker, H. Choset, Continuum robots for medical applications: A survey. *IEEE Trans. Robot.* **31**, 1261–1280 (2015).
40. J. Rodés-Cabau, R. T. Hahn, A. Latib, M. Laule, A. Lauten, F. Maisano, J. Schofer, F. Campelo-Parada, R. Puri, A. Vahanian, Transcatheter therapies for treating tricuspid regurgitation. *J. Am. Coll. Cardiol.* **67**, 1829–1845 (2016).
41. A. Lauten, H. R. Figulla, A. Unbehaun, N. Fam, J. Schofer, T. Doenst, J. Hausleiter, M. Franz, C. Jung, H. Dreger, D. Leistner, B. Alushi, A. Stundl, U. Landmesser, V. Falk, K. Stangl, M. Laule, Interventional treatment of severe tricuspid regurgitation. *Circ. Cardiovasc. Interv.* **11**, e006061 (2018).
42. L. Asmarats, R. Puri, A. Latib, J. L. Navia, J. Rodés-Cabau, Transcatheter tricuspid valve interventions: Landscape, challenges, and future directions. *J. Am. Coll. Cardiol.* **71**, 2935–2956 (2018).
43. K. Matli, A. Mahdi, V. Zibara, C. Costanian, G. Ghanem, Transcatheter tricuspid valve intervention techniques and procedural steps for the treatment of tricuspid regurgitation: a review of the literature. *Open Heart* **9**, e002030 (2022).
44. K. Keller, C. Sinning, A. Schulz, C. Jünger, V. H. Schmitt, O. Hahad, T. Zeller, M. Beutel, N. Pfeiffer, K. Strauch, S. Blankenberg, K. J. Lackner, J. H. Prochaska, E. Schulz, T. Münzel, P. S. Wild, Right atrium size in the general population. *Sci. Rep.* **11**, 22523 (2021).
45. E. S. Gang, B. L. Nguyen, Y. Shachar, L. Farkas, L. Farkas, B. Marx, D. Johnson, M. C. Fishbein, C. Gaudio, S. J. Kim, Dynamically shaped magnetic fields: Initial animal validation of a new remote

electrophysiology catheter guidance and control system. *Circ. Arrhythm. Electrophysiol.* **4**, 770–777 (2011).

46. S. Kumar, J. B. Morton, K. Halloran, S. J. Spence, G. Lee, M. C. Wong, P. M. Kistler, J. M. Kalman, Effect of respiration on catheter-tissue contact force during ablation of atrial arrhythmias. *Heart Rhythm* **9**, 1041–1047.e1 (2012).
47. C. R. Wagner, D. P. Perrin, R. D. Howe, N. Vasilyev, P. J. D. Nido, Force feedback in a three-dimensional ultrasound-guided surgical task, in *14th Symposium on Haptics Interfaces for Virtual Environment and Teleoperator Systems (HAPTICS 2006)* (IEEE, 2006), pp. 43–48
48. S. B. Kesner, R. D. Howe, Robotic catheter cardiac ablation combining ultrasound guidance and force control. *Int. J. Robot. Res.* **33**, 631–644 (2014).
49. N. V. Vasilyev, A. H. Gosline, E. Butler, N. Lang, P. J. Codd, H. Yamauchi, E. N. Feins, C. R. Folk, A. L. Cohen, R. Chen, D. Zurakowski, P. J. D. Nido, P. E. Dupont, Percutaneous steerable robotic tool delivery platform and metal microelectromechanical systems device for tissue manipulation and approximation: Closure of patent foramen ovale in an animal model. *Circ. Cardiovasc. Interv.* **6**, 468–475 (2013).
50. L. Cruddas, G. Martin, C. Riga, Robotic endovascular surgery: Current and future practice. *Semin. Vasc. Surg.* **34**, 233–240 (2021).
51. A. Püschel, C. Schafmayer, J. Groß, Robot-assisted techniques in vascular and endovascular surgery. *Langenbecks Arch. Surg.* **407**, 1789–1795 (2022).
52. M. Mhanna, A. Beran, A. Al-Abdouh, O. Sajdeya, M. Barbarawi, M. Alsaiqali, A. Jabri, A. Al-Aaraj, A. Alharbi, P. Chacko, Steerable versus nonsteerable sheath technology in atrial fibrillation ablation: A systematic review and meta-analysis. *J. Arrhythm.* **38**, 570–579 (2022).
53. M. Singh, C. E. Varela, W. Whyte, M. A. Horvath, N. C. S. Tan, C. B. Ong, P. Liang, M. L. Schermerhorn, E. T. Roche, T. W. J. Steele, Minimally invasive electroceutical catheter for endoluminal defect sealing. *Sci. Adv.* **7**, eabf6855 (2021).
54. E. T. Roche, A. Fabozzo, Y. Lee, P. Polygerinos, I. Friehs, L. Schuster, W. Whyte, A. M. C. Berazaluce, A. Bueno, N. Lang, M. J. N. Pereira, E. Feins, S. Wasserman, E. D. O'Cearbhaill, N. V. Vasilyev, D. J. Mooney, J. M. Karp, P. J. del Nido, C. J. Walsh, A light-reflecting balloon catheter for atraumatic tissue defect repair. *Sci. Transl. Med.* **7**, 306ra149 (2015).
55. A. Ali, D. Dodou, G. Smit, R. Rink, P. Breedveld, Stabilizing interventional instruments in the cardiovascular system: A classification of mechanisms. *Med. Eng. Phys.* **89**, 22–32 (2021).

56. J. J. Zhou, A. Quadri, A. Sewani, Y. Alawneh, R. Gilliland-Rocque, C. Magnin, A. Dueck, G. A. Wright, M. A. Tavallaei, The cathpilot: A novel approach for accurate interventional device steering and tracking. *IEEE/ASME Trans. Mechatron.* **27**, 5812–5823 (2022).
57. L. P. Gaffney, P. M. Loschak, R. D. Howe, A deployable transseptal brace for stabilizing cardiac catheters. *J. Mech. Des. N Y* **140**, 0750031–7500312 (2018).
58. S. B. Kesner, R. D. Howe, Position control of motion compensation cardiac catheters. *IEEE Trans. Robot.* **27**, 1045–1055 (2011).
59. A. Degirmenci, P. M. Loschak, C. M. Tschabrunn, E. Anter, R. D. Howe, Compensation for unconstrained catheter shaft motion in cardiac catheters. *IEEE Int. Conf. Robot. Autom.* **2016**, 4436–4442 (2016).
60. N. U. Nayar, R. Qi, J. P. Desai, Toward the design and development of a robotic transcatheter delivery system for mitral valve implant. *IEEE Trans. Med. Robot. Bionics* **4**, 922–934 (2022).
61. L. Gheorghe, B. J. Rensing, J. A. V. der Heyden, B. Rana, M. C. Post, M. J. Swaans, Tricuspid regurgitation: No longer the ‘forgotten’ valve. *EMJ Cardiol.* **7**, 119–127 (2019).
62. A. Parolari, F. Barili, A. Pilozzi, D. Pacini, Ring or suture annuloplasty for tricuspid regurgitation? A meta-analysis review. *Ann. Thorac. Surg.* **98**, 2255–2263 (2014).
63. J. Gafford, T. Ranzani, S. Russo, A. Degirmenci, S. Kesner, R. Howe, R. Wood, C. Walsh, Toward medical devices with integrated mechanisms, sensors, and actuators via printed-circuit mems. *J. Med. Devices* **11**, 011007 (2017).
64. S. Becker, T. Ranzani, S. Russo, R. J. Wood, Pop-up tissue retraction mechanism for endoscopic surgery, in *IEEE International Conference on Intelligent Robots and Systems* (IEEE, 2017), pp, 920–927.
65. T. Ranzani, S. Russo, N. W. Bartlett, M. Wehner, R. J. Wood, Increasing the dimensionality of soft microstructures through injection-induced self-folding. *Adv. Mater.* **30**, 1–15 (2018).
66. R. Beigel, B. Cercek, H. Luo, R. J. Siegel, Noninvasive evaluation of right atrial pressure. *J. Am. Soc. Echocardiogr.* **26**, 1033–1042 (2013).
67. D. B. Camasão, D. Mantovani, The mechanical characterization of blood vessels and their substitutes in the continuous quest for physiological-relevant performances. a critical review. *Mater. Today Bio* **10**, 100106 (2021).
68. R. J. Gusic, M. Petko, R. Myung, J. W. Gaynor, K. J. Gooch, Mechanical properties of native and ex vivo remodeled porcine saphenous veins. *J. Biomech.* **38**, 1770–1779 (2005).

69. J. Rogatinsky, K. Gomatam, Z. H. Lim, M. Lee, L. Kinnicutt, C. Duriez, P. Thomson, K. McDonald, T. Ranzani, A collapsible soft actuator facilitates performance in constrained environments. *Adv. Intell. Syst.* **4**, 2200085 (2022).
70. T. Ranzani, S. Russo, F. Schwab, C. J. Walsh, R. J. Wood, Deployable stabilization mechanisms for endoscopic procedures, in *Proceedings - IEEE International Conference on Robotics and Automation* (ICRA, 2017), pp. 1125–1131.
71. A. S. Tang, G. A. Wells, M. Talajic, M. O. Arnold, R. Sheldon, S. Connolly, S. H. Hohnloser, G. Nichol, D. H. Birnie, J. L. Sapp, R. Yee, J. S. Healey, J. L. Rouleau, Cardiac-resynchronization therapy for mild-to-moderate heart failure. *N. Engl. J. Med.* **363**, 2385–2395 (2010).
72. A. D. Costa, A. Gate-Martinet, P. Rouffiange, A. Cerisier, A. Nadrouss, L. Bisch, C. Romeyer-Bouchard, K. Isaaz, Anatomical factors involved in difficult cardiac resynchronization therapy procedure: A non-invasive study using dual-source 64-multi-slice computed tomography. *Europace* **14**, 833–840 (2012).
73. A. Noheria, C. V. Desimone, N. Lachman, W. D. Edwards, A. S. Gami, J. J. Maleszewski, P. A. Friedman, T. M. Munger, S. C. Hammill, D. L. Hayes, D. L. Packer, S. J. Asirvatham, Anatomy of the coronary sinus and epicardial coronary venous system in 620 hearts: An electrophysiology perspective. *J. Cardiovasc. Electrophysiol.* **24**, 1–6 (2013).
74. G. Benfari, C. Antoine, W. L. Miller, P. Thapa, Y. Topilsky, A. Rossi, H. I. Michelena, S. Pislaru, M. Enriquez-Sarano, Excess mortality associated with functional tricuspid regurgitation complicating heart failure with reduced ejection fraction. *Circulation* **140**, 196–206 (2019).
75. E. Chorin, Z. Rozenbaum, Y. Topilsky, M. Konigstein, T. Ziv-Baran, E. Richert, G. Keren, S. Banai, Tricuspid regurgitation and long-term clinical outcomes. *Eur. Heart J. Cardiovasc. Imaging* **21**, 157–165 (2019).
76. D. Messika-Zeitoun, P. Verta, J. Gregson, S. J. Pocock, I. Boero, T. E. Feldman, W. T. Abraham, J. A. Lindenfeld, J. Bax, M. Leon, M. Enriquez-Sarano, Impact of tricuspid regurgitation on survival in patients with heart failure: A large electronic health record patient-level database analysis. *Eur. J. Heart Fail.* **22**, 1803–1813 (2020).
77. S. Singh-Gryzbon, A. W. Siefert, E. L. Pierce, A. P. Yoganathan, Tricuspid valve annular mechanics: Interactions with and implications for transcatheter devices. *Cardiovasc. Eng. Technol.* **10**, 193–204 (2019).

78. K. Owais, C. E. Taylor, L. Jiang, K. R. Khabbaz, M. Montealegre-Gallegos, R. Matyal, J. H. Gorman, R. C. Gorman, F. Mahmood, Tricuspid annulus: A three-dimensional deconstruction and reconstruction. *Ann. Thorac. Surg.* **98**, 1536–1542 (2014).
79. K. Emilsson, R. Egerlid, B. Nygren, Tricuspid annulus motion and mitral annulus motion: Anatomical intimacy causing a good correlation? *Exp. Clin. Cardiol.* **10**, 111–115 (2005).
80. M. C. Darnell, J. Y. Sun, M. Mehta, C. Johnson, P. R. Arany, Z. Suo, D. J. Mooney, Performance and biocompatibility of extremely tough alginate/polyacrylamide hydrogels. *Biomaterials* **34**, 8042–8048 (2013).
81. J. Mark, *Physical Properties of Polymers Handbook* (Springer, 2007).
82. B. Faurie, G. Souteyrand, P. Staat, M. Godin, C. Caussin, E. V. Belle, L. Mangin, P. Meyer, N. Dumonteil, M. Abdellaoui, J. Monségu, I. Durand-Zaleski, T. Lefèvre, Left ventricular rapid pacing via the valve delivery guidewire in transcatheter aortic valve replacement. *J. Am. Coll. Cardiol. Intv.* **12**, 2449–2459 (2019).
83. I. Daehnert, C. Rotzsch, M. Wiener, P. Schneider, Rapid right ventricular pacing is an alternative to adenosine in catheter interventional procedures for congenital heart disease. *Heart* **90**, 1047–1050 (2004).
84. R. Scarsini, R. A. Kotronias, G. L. D. Maria, S. Rajasundaram, T. J. Cahill, R. Brown, J. D. Newton, A. P. Banning, R. K. Kharbanda, Routine left ventricular pacing for patients undergoing transcatheter aortic valve replacement. *Struct. Heart* **3**, 478–482 (2019).
85. B. M. Jones, Y. Jobanputra, A. Krishnaswamy, S. Mick, M. Bhargava, B. L. Wilkoff, S. R. Kapadia, Rapid ventricular pacing during transcatheter valve procedures using an internal device and programmer: A demonstration of feasibility. *Catheter. Cardiovasc. Interv.* **95**, 1042–1048 (2020).
86. C. J. Chung, I. George, Emerging transcatheter therapies for tricuspid valve disease. *JTCVS Open* **2**, 14–19 (2020).
87. D. I. Blusztein, R. T. Hahn, New therapeutic approach for tricuspid regurgitation: Transcatheter tricuspid valve replacement or repair. *Front. Cardiovasc. Med.* **10**, 1080101 (2023).
88. M. Mehr, M. Taramasso, C. Besler, T. Ruf, K. A. Connelly, M. Weber, E. Yzeiraj, D. Schiavi, A. Mangieri, L. Vaskelyte, H. Alessandrini, F. Deuschl, N. Brugger, H. Ahmad, L. Biasco, M. Orban, S. Deseive, D. Braun, K. P. Rommel, A. Pozzoli, C. Frerker, M. Näbauer, S. Massberg, G. Pedrazzini, G. H. Tang, S. Windecker, U. Schäfer, K. H. Kuck, H. Sievert, P. Denti, A. Latib, J. Schofer, G. Nickenig, N. Fam, S. von Bardeleben, P. Lurz, F. Maisano, J. Hausleiter, 1-year

outcomes after edge-to-edge valve repair for symptomatic tricuspid regurgitation: Results from the trivalve registry. *J. Am. Coll. Cardiol. Intv.* **12**, 1451–1461 (2019).

89. S. Kodali, R. T. Hahn, M. F. Eleid, R. Kipperman, R. Smith, D. S. Lim, W. A. Gray, A. Narang, S. V. Pislaru, K. Koulogiannis, P. Grayburn, D. Fowler, K. Hawthorne, A. Dahou, S. H. Deo, P. Vandrangi, F. Deuschl, M. J. Mack, M. B. Leon, T. Feldman, C. J. Davidson; CLASP TR EFS Investigators, Feasibility study of the transcatheter valve repair system for severe tricuspid regurgitation. *J. Am. Coll. Cardiol.* **77**, 345–356 (2021).
90. F. Schlotter, M. Miura, K. P. Kresoja, B. Alushi, H. Alessandrini, A. Attinger-Toller, C. Besler, L. Biasco, D. Braun, E. Brochet, K. A. Connelly, S. de Bruijn, P. Denti, R. Estevez-Loureiro, N. Fam, M. Gavazzoni, D. Himbert, E. C. Ho, J. M. Juliard, D. Kalbacher, R. Kaple, F. Kreidel, A. Latib, E. Lubos, S. Ludwig, M. Mehr, V. Monivas, T. M. Nazif, G. Nickenig, G. Pedrazzini, A. Pozzoli, F. Praz, R. Puri, J. Rodés-Cabau, K. P. Rommel, U. Schäfer, J. Schofer, H. Sievert, G. H. L. Tang, H. Thiele, M. Unterhuber, A. Vahanian, R. S. von Bardeleben, M. von Roeder, J. G. Webb, M. Weber, M. G. Wild, S. Windecker, M. Zuber, J. Hausleiter, F. Maisano, M. B. Leon, R. T. Hahn, A. Lauten, M. Taramasso, P. Lurz, Outcomes of transcatheter tricuspid valve intervention by right ventricular function: A multicentre propensity-matched analysis. *EuroIntervention* **17**, e343–e352 (2021).
91. G. Russo, M. Taramasso, D. Pedicino, M. Gennari, M. Gavazzoni, A. Pozzoli, D. Muraru, L. P. Badano, M. Metra, F. Maisano, Challenges and future perspectives of transcatheter tricuspid valve interventions: Adopt old strategies or adapt to new opportunities? *Eur. J. Heart Fail.* **24**, 442–454 (2022).
92. C. M. Yu, G. B. Bleeker, J. W. H. Fung, M. J. Schalij, Q. Zhang, E. E. V. D. Wall, Y. S. Chan, S. L. Kong, J. J. Bax, Left ventricular reverse remodeling but not clinical improvement predicts long-term survival after cardiac resynchronization therapy. *Circulation* **112**, 1580–1586 (2005).
93. F. S. O. Souza, D. M. Braile, R. W. Vieira, S. O. Rojas, N. L. Mortati, A. C. Rabelo, S. A. Oliveira, Technical aspects of lead implantation for left ventricle pacing through the coronary sinus, using anatomic radiology and intracavitary electrography in the cardiac resynchronization therapy. *Braz. J. Cardiovasc. Surg.* **20**, 301–309 (2005).
94. J. H. Gamble, N. Herring, M. Ginks, K. Rajappan, Y. Bashir, T. R. Betts, Procedural success of left ventricular lead placement for cardiac resynchronization therapy: A meta-analysis. *JACC Clin. Electrophysiol.* **2**, 69–77 (2016).

95. H. Mair, J. Sachweh, B. Meuris, G. Nollert, M. Schmoeckel, A. Schuetz, B. Reichart, S. Daebritz, Surgical epicardial left ventricular lead versus coronary sinus lead placement in biventricular pacing. *Eur. J. Cardiothorac. Surg.* **27**, 235–242 (2005).
96. N. Bogale, K. Witte, S. Priori, J. Cleland, A. Auricchio, F. Gadler, A. Gitt, T. Limbourg, C. Linde, K. Dickstein, F. Fruhwald, B. Strohmer, M. Goethals, J. Vijgen, J. N. Trochu, D. Gras, M. Kindermann, C. Stellbrink, K. McDonnald, D. Keane, T. B. Gal, M. Glikson, M. Metra, M. Gasparini, A. Maass, L. Jordaens, M. Alings, A. I. Larsen, S. Færestrand, J. Delgado, L. Mont, H. Persson, H. P. B.-L. Rocca, S. Osswald, I. Squire, J. Morgan, The european cardiac resynchronization therapy survey: Comparison of outcomes between de novo cardiac resynchronization therapy implantations and upgrades. *Eur. J. Heart Fail.* **13**, 974–983 (2011).
97. A. S. Manolis, S. Koulouris, D. Tsiachris, Electrophysiology catheter-facilitated coronary sinus cannulation and implantation of cardiac resynchronization therapy systems. *Hellenic J. Cardiol.* **59**, 26–33 (2018).
98. D. Hofer, A. Breitenstein, Snare technique for coronary sinus cannulation in cardiac resynchronization therapy. *Indian Pacing Electrophysiol. J.* **20**, 293–295 (2020).
99. J. L. D'Arcy, S. Coffey, M. A. Loudon, A. Kennedy, J. Pearson-Stuttard, J. Birks, E. Frangou, A. J. Farmer, D. Mant, J. Wilson, S. G. Myerson, B. D. Prendergast, Large-scale community echocardiographic screening reveals a major burden of undiagnosed valvular heart disease in older people: The oxvalve population cohort study. *Eur. Heart J.* **37**, 3515–3522 (2016).
100. A. L. Axtell, V. Bhambhani, P. Moonsamy, E. W. Healy, M. H. Picard, T. M. Sundt, J. H. Wasfy, Surgery does not improve survival in patients with isolated severe tricuspid regurgitation. *J. Am. Coll. Cardiol.* **74**, 715–725 (2019).
101. Y. Topilsky, J. M. Inojosa, G. Benfari, O. Vaturi, S. Maltais, H. Michelena, S. Mankad, M. Enriquez-Sarano, Clinical presentation and outcome of tricuspid regurgitation in patients with systolic dysfunction. *Eur. Heart J.* **39**, 3584–3592 (2018).
102. O. Stuge, J. Liddicoat, Emerging opportunities for cardiac surgeons within structural heart disease. *J. Thoracic Cardiovasc. Surg.* **132**, 1258–1261 (2006).
103. F. Alqahtani, C. O. Berzingi, S. Aljohani, M. Hijazi, A. Al-Hallak, M. Alkhouli, Contemporary trends in the use and outcomes of surgical treatment of tricuspid regurgitation. *J. Am. Heart Assoc.* **6**, e007597 (2017).

104. B. D. Prendergast, H. Baumgartner, V. Delgado, O. Gérard, M. Haude, A. Himmelmann, B. Iung, M. Leafstedt, J. Lennartz, F. Maisano, E. A. Marinelli, T. Modine, M. Mueller, S. R. Redwood, O. Rörick, C. Sahyoun, E. Saillant, L. Søndergaard, M. Thoenes, K. Thomitzek, M. Tschernich, A. Vahanian, O. Wendler, E. J. Zemke, J. J. Bax, Transcatheter heart valve interventions: where are we? where are we going? *Eur. Heart J.* **40**, 422–440 (2019).

105. N. V. Vasilyev, P. E. Dupont, P. J. D. Nido, Robotics and imaging in congenital heart surgery. *Future Cardiol.* **8**, 285–296 (2012).

106. H. Rafii-Tari, C. J. Payne, G. Z. Yang, Current and emerging robot-assisted endovascular catheterization technologies: A review. *Ann. Biomed. Eng.* **42**, 697–715 (2014).

107. L. Asmarats, M. Taramasso, J. Rodés-Cabau, Tricuspid valve disease: Diagnosis, prognosis and management of a rapidly evolving field. *Nat. Rev. Cardiol.* **16**, 538–554 (2019).

108. M. Arnold, J. Haug, M. Landendinger, Tricuspid annuloplasty: Transcatheter approaches. *Curr. Cardiol. Rep.* **23**, 139 (2021).

109. G. Muntané-Carol, A. Alperi, L. Faroux, E. Bédard, F. Philippon, J. Rodés-Cabau, Transcatheter tricuspid valve intervention: Coaptation devices. *Front. Cardiovasc. Med.* **7**, 139 (2020).

110. M. Taramasso, R. T. Hahn, H. Alessandrini, A. Latib, A. Attinger-Toller, D. Braun, E. Brochet, K. A. Connelly, P. Denti, F. Deuschl, A. Englmaier, N. Fam, C. Frerker, J. Hausleiter, J. M. Juliard, R. Kaple, F. Kreidel, K. H. Kuck, S. Kuwata, M. Ancona, M. Malasa, T. Nazif, G. Nickenig, F. Nietlispach, A. Pozzoli, U. Schäfer, J. Schofer, R. Schueler, G. Tang, A. Vahanian, J. G. Webb, E. Yzeiraj, F. Maisano, M. B. Leon, The international multicenter trivalve registry: Which patients are undergoing transcatheter tricuspid repair? *JACC: Cardiovascular Interventions* **10**, 1982–1990 (2017).

111. J. Mesnier, A. Alperi, V. Panagides, E. Bédard, E. Salaun, F. Philippon, J. Rodés-Cabau, Transcatheter tricuspid valve interventions: Current devices and associated evidence. *Prog. Cardiovasc. Dis.* **69**, 89–100 (2021).

112. D. Kolte, S. Elmariah, Current state of transcatheter tricuspid valve repair. *Cardiovasc. Diagn. Ther.* **10**, 89–97 (2020).

113. I. R. J. Webster, B. A. Jones, Design and kinematic modeling of constant curvature continuum robots: A review. *Robot. Res.* **29**, 1661–1683 (2010).

114. P. Rao, Q. Peyron, S. Lilge, J. Burgner-kahrs, How to model tendon-driven continuum robots and benchmark modelling performance. *Front. Robot. AI* **7**, 630245 (2015).

115. T. M. Vesely, T. K. Pilgram, Angioplasty balloon inflation pressures during treatment of hemodialysis graft-related stenoses. *J. Vasc. Interv. Radiol.* **17**, 623–628 (2006).
